# Intrapartum uterine activity and neonatal outcomes: a systematic review

**DOI:** 10.1186/s12884-020-03219-w

**Published:** 2020-09-12

**Authors:** Adam J. Reynolds, Michael P. Geary, Breda C. Hayes

**Affiliations:** 1 Department of Neonatology, The Rotunda Hospital, Dublin, Ireland; 2 Department of Obstetrics and Gynaecology, The Rotunda Hospital, Dublin, Ireland

**Keywords:** Labour, Intrapartum, Fetal monitoring, Tachysystole, Uterine activity, Uterine contraction

## Abstract

**Background:**

Increased uterine activity (UA) may not allow adequate recovery time for foetal oxygenation.

**Methods:**

The aim of the study was to determine if increased UA during labour is associated with an increased risk of either short- or long-term neurological injury in term neonates, or with neonatal proxy measures of intrapartum hypoxia-ischemia. MEDLINE, CINAHL, and ClinicalTrials.gov were searched using the following terms: uterine activity, excessive uterine activity, XSUA, uterine hyperstimulation, and tachysystole. Any study that analysed the relationship between UA during term labour and neurological outcomes/selected proxy neurological outcomes was eligible for inclusion. Outcomes from individual studies were reported in tables and presented descriptively with odds ratios (OR) and 95% confidence intervals (CI) for dichotomous outcomes and means with standard deviations for continuous outcomes. Where group numbers were provided, ORs and their CIs were calculated according to Altman.

**Main results:**

Twelve studies met the inclusion criteria. Seven studies featured umbilical artery pH as an individual outcome. Umbilical artery base excess and Apgar scores were both reported as individual outcomes in four studies. No study examined long term neurodevelopmental outcomes and only one study reported on encephalopathy as an outcome. The evidence for a relationship between UA and adverse infant outcomes was inconsistent. The reported estimated effect size varied from non-existent to clinically significant.

**Conclusions:**

There is some evidence that increased UA may be a non-specific predictor of depressed neurological function in the newborn, but it is inconsistent and insufficient to support the conclusion that an association generally exists.

## Background

The rationale for the proposed link between excessive uterine activity (UA) and fetal hypoxia is based on physiological studies of the haemodynamic changes that occur in the utero-placental and fetal circulations during a contraction. As a uterine contraction progresses, the amount of oxygenated blood delivered to the placenta decreases. Thus, the lowest fetal oxygen saturation percentage values are found towards the end of a contraction and take some time to recover. Increased contraction frequencies may not allow adequate recovery time and may result in progressive reductions in fetal oxygen levels [1–3].

There is evidence that tocolytic medications may improve fetal heart rate (FHR) abnormalities when used while emergency delivery is pending [4]. Compared to emergent delivery, tocolysis for fetal distress may improve umbilical artery (UmA) base excess (BE) values and reduce neonatal intensive care unit admission at the expense of increasing caesarean delivery [5]. A 2018 Cochrane review found several studies which showed an improvement in fetal wellbeing in response to tocolysis but concluded that, given the small sample sizes involved, “the clinical significance is unclear” [6].

Current guidelines are based on the opinion that when there is an abnormal FHR pattern in association with frequent uterine contractions, increased UA may cause fetal hypoxia [7]. The 2014 American College of Obstetricians and Gynaecologists (ACOG) guidelines, Neonatal Encephalopathy and Neurologic Outcome, define tachysystole (TS) as more than five contractions in 10 min averaged over a 30-min window, and state that TS should be treated whenever it is associated with recurrent FHR decelerations or if oxytocin is being administered [8]. The UK National Institute for Health and Care Excellence guidelines on labour management state that where the FHR pattern is suspicious or pathological any uterine hyperstimulation should be corrected [9]. The authors are unaware of any existing guidelines which address TS in unaugmented labour without FHR abnormalities.

The aim of this systematic review is to determine if increased UA during labour is associated with an increased likelihood of either short- or long-term neurological injury in term neonates, or with proxy neonatal measures of intrapartum hypoxia-ischemia.

## Methods

The study protocol was registered with PROSPERO (CRD4201705258) and followed the Preferred Reporting Items for Systematic Reviews and Meta-Analyses (PRISMA) statement.

### Search strategy

We searched MEDLINE, CINAHL, and ClinicalTrials.gov using terms related to UA (“uterine activity”, “excessive uterine activity”, “XSUA”, “uterine hyperstimulation”, and “tachysystole”). Due to concern about the impact of changes in clinical practice over time, the search was limited to articles published after January 1^st^, 1996. Reference lists of relevant papers were checked to identify further papers for consideration. The results were de-duplicated using COVIDENCE (Covidence systematic review software, Veritas Health Innovation Ltd., Melbourne, Australia). The search was repeated prior to final analysis.

### Eligibility criteria

Eligibility criteria were pre-determined. Based on the anticipated heterogeneity and small size of the evidence base, we opted for permissive eligibility criteria. Any study that analysed the relationship between UA during term labour and neurological outcomes or selected proxy outcomes was eligible. The proxy outcomes were selected because they are commonly used indicators of intrapartum hypoxia-ischemia [10]. Based on incomplete reporting, conference abstracts were excluded. Studies retrieved were screened by one author and potentially eligible studies were then assessed by two team members before a final inclusion decision.

### Data extraction and evidence summary

A standardised form was used to extract data. Missing data was requested from authors and included if returned. Outcomes from individual studies were reported in tables and presented descriptively with odds ratios (OR) and 95% confidence intervals (CI) for dichotomous outcomes and means with standard deviations (SD) for continuous outcomes where available. Where group numbers were provided, ORs and their CIs were calculated according to Altman [11]. Results adjusted based on multivariate analysis were included as reported. A post-hoc analysis of the relationship between uterine activity and both FHR patterns and delivery mode was also performed.

### Quality assessment

The risk of bias was independently assessed by both reviewers using The Scottish Intercollegiate Guideline Network (SIGN) critical appraisal checklists but was not used to exclude any results.

### Funding

Funding was provided by The National Women and Infants Health Programme (Ireland) and the Rotunda Foundation (Registered Charity Number. 20079529).

## Results

### Search results and study selection

**Fig. 1:**
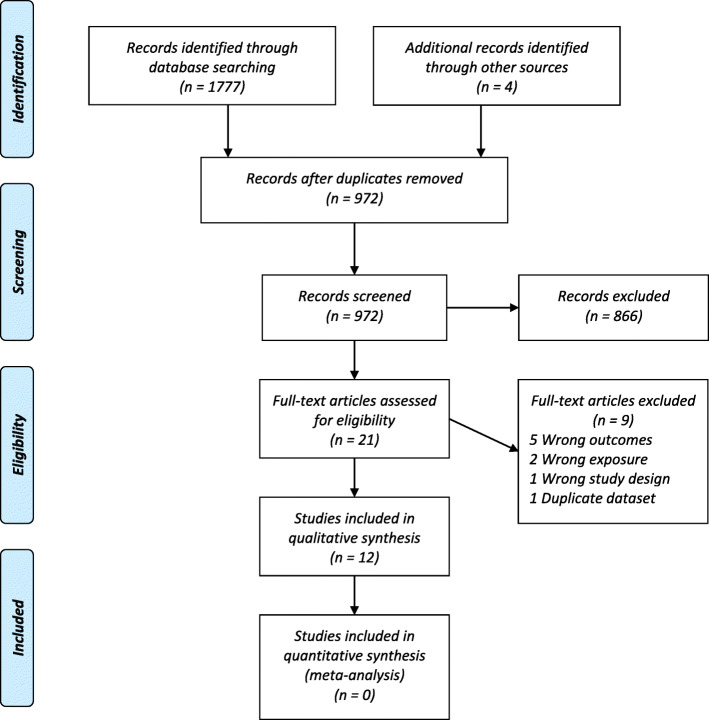
PRISMA diagram

Our search retrieved 1777 citations from MEDLINE, CINAHL. (Table S1) Four additional articles were identified after review of references. The search result details are specified in supplementary material. A search of ClinicalTrials.gov did not reveal any unpublished but otherwise eligible studies. Two conference abstracts with possibly eligible results were identified but not included owing to the incomplete nature of the reports [12, 13]. After screening of abstracts, 21 articles were selected for full-text review. Of these, nine were excluded [3, 14–21]. (Fig. 1).

### Study characteristics

The characteristics of each included study are summarized in Table S2. Twelve studies met the inclusion criteria [22–33]. In five [22, 23, 26, 31, 32], the primary analysis was grouped by exposure i.e. UA. In three [24, 30, 33], cases were identified and then selectively matched to controls. For the other nine studies, all eligible patients were recruited regardless of outcome (median subject number: 1433 [interquartile range: 720–8008, range: 430–50,335]). In three [23, 25, 27] data was collected prospectively with the primary purpose being to compare UA to fetal outcomes. In two [31, 32], data was collected prospectively but the included studies were secondary analyses. The data collected pertained to labours from 1993 to 2015. Subjects were recruited in North America (7 studies), Sweden, Turkey, Italy, The Netherlands, and Ireland (1 study each).

Included labours were exclusively spontaneous and unaugmented [26], exclusively induced/augmented [23, 25, 27, 32], or mixed [22, 24, 28–31, 33].

One study [29] exclusively used measurements from intra-uterine pressure catheters (IUPC). Three studies [27, 32, 33] used external tocography data only. Two studies [23, 30] reported using IUPC and external tocography but mainly external measurements. Six studies [22, 24–26, 28, 31] did not specify the measurement technology.

Three studies [28–30] used automated analysis. Four studies [22, 26, 27, 33] used documentation recorded during labour by the attending midwives The remainder [23–25, 31, 32] reported retrospectively analysed recordings, the interpretation of which was stated to be blinded in all except one [32].

The period of labour analysed varied significantly between studies. Three [26, 27, 33] did not explicitly state the period included. Two [25, 32] analysed the entire period from induction to delivery. One [24] analysed data from the first 4 h after induction. The remainder analysed a period leading up to delivery (Bakker: last hour of 1st stage and total 2nd stage, Hamilton: 4 h, Smith: ≤4 h, Heuser: ≥2 h, Jonsson: 2 h, Palanisamy: 1 h) [22, 24, 29–31].

Eleven studies featured contraction rate as an individual exposure, with one [27] reporting rate only as part of a combined exposure with contraction duration. All studies apart from Bakker et al. reported contraction rate as either a dichotomised or categorised variable. Two studies [29, 30] included contraction rate as a continuous variable. All studies reported dichotomised outcomes only apart from one study [27] which reported UmA pH and BE as continuous measures.

Six of the ten included studies published after 2008 adhered precisely or closely to the ACOG 2008 definition of TS i.e. more than five contractions in 10 min, averaged over a 30-min window. Of the four that did not follow the ACOG definition: Hayes et al. referred to 15-min contraction rates > 7, Mutlu Meydanli et al. reported a composite exposure which included the ACOG definition along with any contraction longer than 2-min, Bofill et al. required two consecutive 10-min windows with ≥6 contractions each, and Stewart et al. reported the maximum 10-min contraction rate [23, 25, 32, 33].

Two studies [23, 29] included non-composite exposures besides contraction rate. Stewart et al. reported the number of contractions > 120 s. Bakker et al. reported on relaxation time as well as contraction duration, amplitude, and surface (mean and total), as well as Montevideo units, and active planimeter units (mean).

### Risk of bias within studies

Of the twelve included studies, three were classed as at high risk of bias, six were classed as at medium risk, and three were classed as at low risk. (Tables S3-S14).

### Synthesis of results

The results of the individual studies are summarized in Table S15.

#### Neurodevelopmental outcome

No studies were found which reported neurodevelopmental outcomes in relation to UA.

#### Hypoxic ischemic encephalopathy

One study reported hypoxic-ischemic encephalopathy (HIE) as an outcome. In Hayes et al. contraction rates greater than 7 in 15 min were associated with an increased risk of neonatal encephalopathy (TS: 87/187 [46.5%], No TS: 120/457 [26.3%], OR: 2.44 [95% CI: 1.71–3.48], aOR: 2.07 [95% CI: 1.13 to 3.81]) [33].

#### Umbilical artery pH

Seven studies featured UmA pH as an individual outcome. Three rejected and four retained the null hypothesis that UA is not associated with UmA pH levels. Jonsson et al. compared cases with UmA pH < 7.05 to controls with pH ≥ 7.05 and 5-min Apgar scores ≥5 [24]. TTS in the last 2 hours before delivery was found to be associated with the likelihood of an adverse outcome (TS: 84/127 [66.1%], No TS: 132/469 [28.1%], OR: 4.99 [95% CI: 3.28–7.58]). Mutlu Meydanli et al. found that TS was associated with a 7.1 (95% CI: 1.3–38.7) times increase in the relative risk of a UA pH ≤7.15 in women after misoprostol induction (Bishop’s score ≤ 4) [25]. Bakker et al. found that UmA pH levels ≤7.11 were associated with higher contractions per 10-min as measured by intrauterine pressure catheters both in the first (mean: 5.0 [SD: 0.7] versus 4.8 [0.7], *p* = 0.006) and second stages (mean: 5.5 [SD: 0.9] versus 5.2 [0.9], *p* = 0.002) [29]. Bofill et al. reported that TS after cervical ripening was not associated with mean UmA pH levels (≥3 episodes: 7.22 [SD: 0.08], No episodes: 7.22 [SD: 0.08], *p* = 0.435), or with the likelihood of an UmA pH < 7.0 (TS: 3/131 [2.3%], No TS: 8/631 [1.3%], OR: 1.83 [95% CI: 0.48–6.97]). Stewart et al. reported that the maximum contraction rate in the first 4 hours after induction was not associated with an increased risk of acidosis (≤4: 1/152 [1%], 5: 4/179 [2%], 6: 2/134 [1%], ≥7: 6/119 [5%], p for trend = 0.06, OR for > 5: 2.20 [95% CI: 0.71–6.81]). Mussi et al. found that the presence of TS or prolonged contractions was not linearly correlated with UmA pH (r = 0.006, *p* = 0.8) [27]. Palanisamy et al. found that TS in the last hour of labour was not associated with UmA pH levels (TS: 9/513 [1.8%], No TS: 140/8067 [1.7%], OR: 1.01 [95% CI: 0.51–2.00]) [31].

#### Umbilical Artery Base excess

Four studies tested whether UmA BE was linked to UA with two rejecting the null hypothesis. Mussi et al. found that the presence of TS or prolonged contractions was not linearly correlated with the UmA BE (r = 0.15, *p* = 0.07). Hamilton et al. compared labours with UA BE <-12 to labours with UA BE >-8. The found that TS was associated with acidaemia (TS: 138/1215 [11.4%], No TS: 178/2105 [8.5%], OR: 1.39 [95% CI: 1.10–1.75]) [30]. In those with TS, the duration of TS was not associated with metabolic acidosis. Bofill et al. found that labours with ≥3 20-min episodes of TS did not have lower mean BE values than labours without TS (mean BE: -6.81 [SD: 4.18] versus − 6.57 [4.0], *p* = 0.535). Palanisamy et al. reported that TS in the last hour before delivery was more common in labours with a BE ≤-8 (TS: 33/513 [6.4%], No TS: 315/8067 [3.9%], OR: 1.69 [95% CI: 1.17–2.45]).

#### Umbilical artery lactate

One study reported UmA lactate values. Palanisamy et al. observed that UmA lactate values ≥4 were more common when the last hour before delivery was complicated by TS (TS: 173/513 [33.7%], No TS: 2032/8067 [25.2%], OR: 1.51 [95% CI: 1.25–1.83]).

#### Apgar score

Four of nine cohort studies included Apgar score as an individual outcome. One rejected and three retained the null hypothesis. Heuser at al. reported that documentation of > 5 contractions in 10 min averaged over 30 min was associated with an increased rate of 5-min Apgar scores < 7 (TS: 61/5363 [1.1%], No TS: 336/44972 [0.7%], OR: 1.53 [95% CI: 1.16–2.01]) [22]. Stewart et al. reported that maximum contraction rate was not associated with an increased risk of a five-minute Apgar ≤3 (≤4: 0/152 (0%), 5: 0/134 (0%), 6: 1/179 (1%), ≥7: 0/119 (0%), p for trend = 0.86) [23]. Ahmed at al. found that TS (> 5 contractions in 10 min averaged over a 30-min window) was not associated with an Apgar score < 7 at 5 min (No TS: 14/7118 [0.2%], TS: 2/890 [0.2%], *p* = 0.897) [26]. Bofill et al. reported that TS was not associated with median 5-min Apgar scores (≥3 TS episodes: 9 [IQR: 8–9], No TS: 9 [IQR: 8–9], *p* = 0.502) or with the risk of 5-min Apgar scores ≤3 (≥3 TS episodes: 0/131 [0.0%], No TS: 3/631 [0.5%]) [32].

#### Composite outcomes

Two studies featured composite outcomes which were composed of eligible outcomes but could not be separated based on available data. Smith et al. compared cases with an UmA BE ≤10 mmol/L or a 5-min Apgar score ≤ 6 to controls without those characteristics. Using automated analysis of external tocography recordings from up to 4 h before delivery, they found that TS regardless of duration was not more common in cases than controls (No TS: 66/5095 [1.3%], Any TS: 11/1139 [1.0%], *p* = 0.45) [28]. Mussi et al. found that TS or prolonged contractions were not associated with either a UmA pH < 7.1 or a UmA BE >-10 (No TS: 11/339 [3.2%], TS: 5/91 [5.5%], *p* = 0.35).

**Fig. 2:**
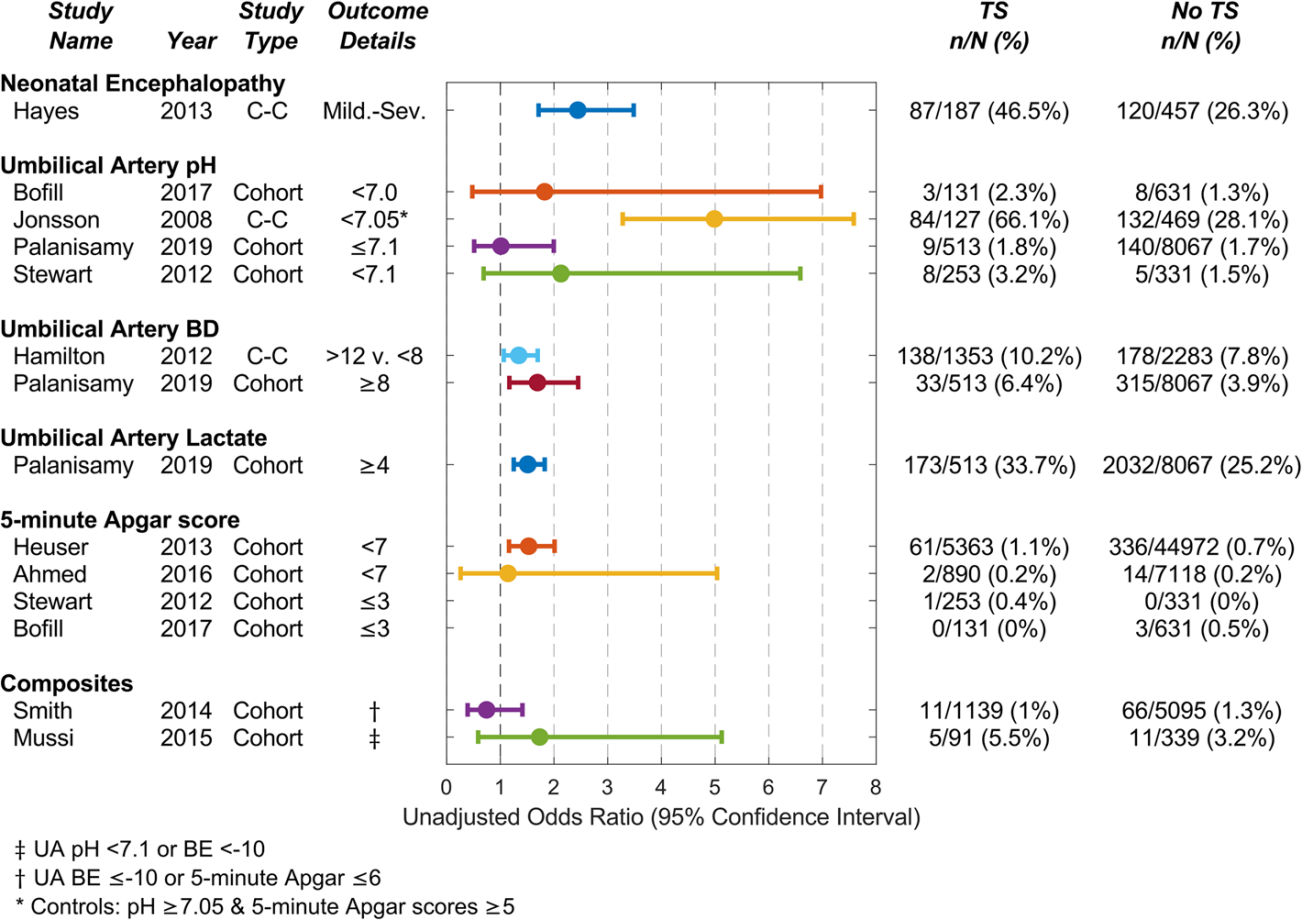
Unadjusted odds ratios (OR) for dichotomous outcomes in the presence of tachysystole (TS). Case control (C-C) study = selective recruitment based on outcome. BD = Base deficit. BD and lactate are expressed in mmol/L. n = number with adverse outcome. N = total group number. Apgar OR not calculable for Stewart and Bofill

Figure 2 shows unadjusted odds ratios for each dichotomous outcome in the presence of TS. Mutlu Meydanli et al. was excluded from the graph as group numbers were not reported.

#### Fetal heart rate assessments

Three of the twelve included studies compared FHR patterns in labours with TS to those without TS. All rejected the null hypothesis. Stewart et al. reported that the maximum 10-min contraction rate was related to the presence of variable, late, or prolonged decelerations (Any decelerations: ≤4: 61/152 [40%], 5: 88/179 [49%], 6: 77/134 [57%], ≥7: 69/119 [58%] p for trend < 0.0.001, OR for > 5: 1.67 [95% CI: 1.20–2.32]). Ahmed et al. found that the rate of non-reassuring FHR (NRFHR) traces in labours with TS was higher than those labours without TS (TS: 38/890 [4.3%], no TS: 179/7118 [2.5%], OR: 1.73 [95% CI: 1.21–2.47]). Bofill et al. found higher rates of NRFHR patterns with TS compared to without (≥3 TS episodes: 28/131 [21%], No episodes: 91/631 [14%], OR: 1.61 [95% CI: 1.01–2.59]).

#### Uterine activity and delivery methods

Three of the included studies reported on TS in relation to caesarean delivery for any indication. None found a statistically significant association. Heuser at al. reported that TS for any half hour period in the last 2 hours of labour was not associated with caesarean delivery (TS: 670/5363 [12.5%], No TS: 5240/44972 [11.7%], OR: 1.08 [95% CI: 0.99–1.18]). Stewart at al. reported that TS in the first 4 hours after induction was not associated with caesarean delivery (TS: 43/253 [17.0%], No TS: 66/331 [19.9%], OR: 0.82 [95% CI: 0.54–1.26]). Bofill et al. found that TS at any stage after induction was not associated with caesarean delivery (TS: 37/131 [28.2%], No TS: 215/631 [34.1%], OR: 0.76 [95% CI: 0.50–1.15]).

Two studies reported data on caesarean delivery for FHR trace findings. Ahmed et al. found TS in spontaneous unaugmented labours to be associated with an increased risk of caesarean delivery for NRFHR traces (TS: 58/890 [6.5%], No TS: 318/7118 [4.5%], OR: 1.49 [95% CI: 1.12–1.99]). Mutlu Meydanli et al. reported a statistically insignificant increase in the risk of caesarean delivery for NRFHR traces (RR: 2.4 [95% CI: 0.6–9.4]).

Heuser et al. found that TS was associated with an increased likelihood of operative vaginal delivery (TS: 682/5363 [12.7%], No TS: 3491/44972 [7.8%], OR: 1.73 [95% CI: 1.59–1.89]).

#### Results for exposures other than contraction rate

With regard to the last hour of the first stage, Bakker at al. found that UmA pH < 7.11 was associated with average relaxation times (cases: 51 s ± 23, controls: 63 s ± 35, *p* < 0.001), average Montevideo units (cases: 261 ± 86, controls: 236 ± 97, *p* = 0.02), and average active planimeter units (cases: 9014 ± 2461, controls: 8379 ± 2740, *p* = 0.04), but not with average contraction duration (cases: 87 s ± 9, controls: 87 s ± 10, *p* = 0.68), average contraction amplitude (cases: 54 mmHg ± 16, controls: 51 mmHg ± 19, *p* = 0.165), or average contraction surface (cases: 1875 mmHg*s ± 555, controls: 1798 ± 600, *p* = 0.26). Similar results were reported for the second stage.

## Conclusions

### Main finding

The evidence for a relationship between UA and adverse infant outcomes is inconsistent. The reported effect sizes vary from small and not statistically significant to highly clinically significant.

### Strengths and limitations

#### Individual studies

Only one study reported direct measures of neurological function in neonates and no study reported long-term neurological outcomes. These interactions are inherently difficult to assess because adverse outcomes of these types are rare and often require long-term follow-up.

As pre-specified in our protocol, NICU admission was excluded as an outcome. Admission to the NICU was reported either as an individual or as part of a composite outcome in three of the included studies [22, 26, 31]. The group of babies admitted to NICU is heterogenous and likely significantly composed of babies conditions not associated with neurological function.

One study explicitly excluded neonates with encephalopathy, potentially introducing significant selection bias. Selection criteria for studies of intrapartum monitoring should allow for the inclusion of labours with negative outcomes including HIE and fetal death. The remaining studies reported proxy outcomes used as measures of intrapartum hypoxia-ischemia i.e. umbilical cord gas values or Apgar scores. These measures indicate an increased risk of neurological injury but are acknowledged to be decidedly non-specific predictors of long-term outcome [34].

None of the included articles reported sample size calculations. Since the relevant adverse outcomes are rare and assuming modest estimated effect sizes, several of the included studies were underpowered for the variables of interest to this review.

When trying to establish a causative relationship, controlling for confounding variables through multivariate analysis should be based on a model of the mechanisms of the purported relationships between the exposure, the outcome, and the potential confounder [35]. Oxytocin and nulliparity are both associated with increased UA [14, 36]. Therefore, in a study of UA and neonatal outcomes, it is not usually appropriate to control for these factors since, according to the hypothesis, they may be causally related to the outcome via their effect on uterine activity. Some included studies e.g. Heuser et al. reported multivariate analyses which included these variables.

Chorioamnionitis, oligohydramnios, pre-eclampsia and the duration of labour have all been associated with both increased UA and adverse neonatal outcomes [22, 26, 37, 38]. For the most part, the connection between these factors and neonatal outcomes is not plausibly via their effect on tachysystole. Therefore, it may be appropriate to control for these factors when analysing the influence of UA on neonatal outcomes. They were not considered in the included studies.

#### Review level

The permissive eligibility criteria for this review allowed a comprehensive survey of the literature. Due to the significant heterogeneity of the studies in terms of overall design, as well as the types and definitions of exposures and outcomes, metanalysis was precluded by this approach. However, given the disparate nature and small size of the evidence base, valid metanalysis would not have been possible even with a narrowly focused review.

The assessment of bias in the individual studies was limited. Current tools for assessing bias in non-interventional studies are not robust [39].

Publication bias is common in systematic reviews and may particularly affect observational studies [40]. Since publication bias is difficult to assess accurately and owing to the heterogeneity of the included studies, no formal assessment was attempted. Study registration and replication have been proposed as solutions to publication bias and selective reporting in observational studies [41, 42]. None of the included trials had protocols registered at ClinicalTrials.gov. No replication studies were found.

### Interpretation

Most commonly UA was defined in terms of rate only, as a dichotomous variable and without regard to labour progress. This model is partly based on unavoidable limitations of external tocography but is nonetheless a reductive view of UA which may obscure the effect of increased UA on neonatal outcomes.

The aim of this review was not to assess the impact of UA levels on FHR patterns. However, it is important to consider how interventions for FHR abnormalities might affect the relationship between UA and neonatal outcomes. Three included studies reported increased rates of FHR pattern abnormalities in labours with TS and another [22] reported worsening of FHR traces to be temporally related to TS. In Bofill et al. labours with TS were more likely to result in caesarean delivery for FHR abnormalities. If increased UA leads to fetal distress, interventions for fetal distress such as caesarean delivery might lessen the impact of increased UA and the observed effect on outcome could be weakened. Therefore, differences in the management of TS and/or fetal distress are among the possible explanations for the disparity in reported results. Future studies should report on delivery methods and their indications, as well as any intrauterine resuscitation administered so that these measures can be taken into account.

As an individual marker, it is unlikely that UA can accurately predict the condition of a fetus after delivery. It is possible that increased UA is not on its own typically sufficient to produce significant fetal hypoxia-ischemia but that, in concert with other factors such as placental insufficiency or prolonged labour, it may contribute to adverse neonatal outcomes.

Based on current evidence, which is limited, tachysystole is common and mostly does not result in neonatal complications. There is inconsistent evidence to support the hypothesis that increased UA is associated with neonatal markers of intrapartum hypoxia-ischemia and depressed neurological function in the newborn.

## Supplementary information

**Additional file 1: PRISMA Checklist.**

**Additional file 2: Supplementary Tables.** detailing the search results, the characteristics of the included studies, the risk of bias assessments, the results of the individual included studies, and the data extraction template.

## Data Availability

All data generated or analysed during this study are included in this published article and its supplementary information files.

## Abbreviations

ACOG: American College of Obstetricians and Gynecologists
BE: Base Excess
C-C: Case-control Study
CI: Confidence Interval
FHR: Fetal Heart Rate
HIE: Hypoxic-Ischemic Encephalopathy
OR: Odds Ratio
PRISMA: Preferred Reporting Items for Systematic Reviews and Meta-Analyses
SD: Standard Deviation
SIGN: Scottish Intercollegiate Guideline Network
TS: Tachysystole
UA: Uterine Activity
UmA: Umbilical Artery
