# Supplementary material for: Intrapartum uterine activity and neonatal outcomes: a systematic review
Source: BMC Pregnancy Childbirth. 2020 Sep 12;20:532. doi: 10.1186/s12884-020-03219-w (PMC7488697; doi:10.1186/s12884-020-03219-w)
Supplement: Supplementary file 1 — Additional file 1: PRISMA Checklist. [file 12884_2020_3219_MOESM1_ESM.docx]

## **Prisma 2009 Checklist**^[[1]](#endnote-2)^

| **Section/topic** | **#** | **Checklist item** | **Reported on page #, section heading** |
| --- | --- | --- | --- |
| **TITLE** | | |  |
| Title | 1 | Identify the report as a systematic review, meta-analysis, or both. | Title page |
| **ABSTRACT** | | |  |
| Structured summary | 2 | Provide a structured summary including, as applicable: background; objectives; data sources; study eligibility criteria, participants, and interventions; study appraisal and synthesis methods; results; limitations; conclusions and implications of key findings; systematic review registration number. | Page 2, Abstract |
| **INTRODUCTION** | | |  |
| Rationale | 3 | Describe the rationale for the review in the context of what is already known. | Page 4, Background |
| Objectives | 4 | Provide an explicit statement of questions being addressed with reference to participants, interventions, comparisons, outcomes, and study design (PICOS). | Page 4, Background |
| **METHODS** | | |  |
| Protocol and registration | 5 | Indicate if a review protocol exists, if and where it can be accessed (e.g., Web address), and, if available, provide registration information including registration number. | Page 5, Methods |
| Eligibility criteria | 6 | Specify study characteristics (e.g., PICOS, length of follow-up) and report characteristics (e.g., years considered, language, publication status) used as criteria for eligibility, giving rationale. | Page 5, Eligibility Criteria |
| Information sources | 7 | Describe all information sources (e.g., databases with dates of coverage, contact with study authors to identify additional studies) in the search and date last searched. | Page 5, Search Strategy  +  Page S1, Search Results |
| Search | 8 | Present full electronic search strategy for at least one database, including any limits used, such that it could be repeated. | Page S1,  Search Results |
| Study selection | 9 | State the process for selecting studies (i.e., screening, eligibility, included in systematic review, and, if applicable, included in the meta-analysis). | Page 5, Eligibility Criteria  +  Page 6, Quality Assessment |
| Data collection process | 10 | Describe method of data extraction from reports (e.g., piloted forms, independently, in duplicate) and any processes for obtaining and confirming data from investigators. | Page 5, Data Extraction and Evidence Summary  +  Page S32, Data Extraction Template |
| Data items | 11 | List and define all variables for which data were sought (e.g., PICOS, funding sources) and any assumptions and simplifications made. | Page S32, Data Extraction Template |
| Risk of bias in individual studies | 12 | Describe methods used for assessing risk of bias of individual studies (including specification of whether this was done at the study or outcome level), and how this information is to be used in any data synthesis. | Page 6, Quality Assessment +  Page S4, Risk of Bias Assessments |
| Summary measures | 13 | State the principal summary measures (e.g., risk ratio, difference in means). | Page 5, Data Extraction and Evidence Summary |
| Synthesis of results | 14 | Describe the methods of handling data and combining results of studies, if done, including measures of consistency (e.g., I^2^) for each meta-analysis. | Page 5, Data Extraction and Evidence Summary |
| Risk of bias across studies | 15 | Specify any assessment of risk of bias that may affect the cumulative evidence (e.g., publication bias, selective reporting within studies). | Page 13, Review Level |
| Additional analyses | 16 | Describe methods of additional analyses (e.g., sensitivity or subgroup analyses, meta-regression), if done, indicating which were pre-specified. | Page 5, Data Extraction and Evidence Summary |
| **RESULTS** | | |  |
| Study selection | 17 | Give numbers of studies screened, assessed for eligibility, and included in the review, with reasons for exclusions at each stage, ideally with a flow diagram. | Page 6, Search Results and Study Selection  +  Figure 1 |
| Study characteristics | 18 | For each study, present characteristics for which data were extracted (e.g., study size, PICOS, follow-up period) and provide the citations. | Page 6, Study Characteristics  +  Page S2, Study Characteristics |
| Risk of bias within studies | 19 | Present data on risk of bias of each study and, if available, any outcome level assessment (see item 12). | Page 8, Risk of Bias Within Studies,  +  Page S4, Risk of Bias Assessments |
| Results of individual studies | 20 | For all outcomes considered (benefits or harms), present, for each study: (a) simple summary data for each intervention group (b) effect estimates and confidence intervals, ideally with a forest plot. | Page 8, Synthesis of Results  +  Page S28, Results of Individual Studies |
| Synthesis of results | 21 | Present results of each meta-analysis done, including confidence intervals and measures of consistency. | Not applicable |
| Risk of bias across studies | 22 | Present results of any assessment of risk of bias across studies (see Item 15). | Not applicable |
| Additional analysis | 23 | Give results of additional analyses, if done (e.g., sensitivity or subgroup analyses, meta-regression [see Item 16]). | Page 10, Fetal Heart Rate Assessments  +  Page 11, Uterine Activity and Delivery Methods |
| **DISCUSSION** | | |  |
| Summary of evidence | 24 | Summarize the main findings including the strength of evidence for each main outcome; consider their relevance to key groups (e.g., healthcare providers, users, and policy makers). | Page 12, Main Finding |
| Limitations | 25 | Discuss limitations at study and outcome level (e.g., risk of bias), and at review-level (e.g., incomplete retrieval of identified research, reporting bias). | Page 13, Strengths and Limitations |
| Conclusions | 26 | Provide a general interpretation of the results in the context of other evidence, and implications for future research. | Page 13, Interpretation |
| **FUNDING** | | |  |
| Funding | 27 | Describe sources of funding for the systematic review and other support (e.g., supply of data), role of funders for the systematic review. | Page 3, Funding |

#

# Supplemental Material Bibliography

1. Kunz MK, Loftus RJ, Nichols AA. Incidence of Uterine Tachysystole in Women Induced with Oxytocin. Journal of Obstetric, Gynecologic & Neonatal Nursing. 2013;42(1):12-8.

1. From: Moher D, Liberati A, Tetzlaff J, Altman DG, The PRISMA Group (2009). Preferred Reporting Items for Systematic Reviews and Meta-Analyses: The PRISMA Statement. PLoS Med 6(7): e1000097. doi:10.1371/journal.pmed1000097. For more information, visit: <http://www.prisma-statement.org>. [↑](#endnote-ref-2)
