# Supplementary material for: Intrapartum uterine activity and neonatal outcomes: a systematic review
Source: BMC Pregnancy Childbirth. 2020 Sep 12;20:532. doi: 10.1186/s12884-020-03219-w (PMC7488697; doi:10.1186/s12884-020-03219-w)
Supplement: Supplementary file 2 — Additional file 2: Supplementary Tables. detailing the search results, the characteristics of the included studies, the risk of bias assessments, the results of the individual included studies, and the data extraction template. [file 12884_2020_3219_MOESM2_ESM.docx]

# Supplemental Material

## Search Results

Table S1. Search strategy and results

| Database | Search Type | Term(s) | Start Date | Date of search | Results |
| --- | --- | --- | --- | --- | --- |
| Ovid Medline (R) | Basic Search | Uterine activity | 1996 | 08/12/2016 | 511 |
| Ovid Medline (R) | Basic Search | Excessive uterine activity | 1996 | 08/12/2016 | 511 |
| Ovid Medline (R) | Basic Search | XSUA | 1996 | 08/12/2016 | 1 |
| Ovid Medline (R) | Basic Search | Uterine hyperstimulation | 1996 | 08/12/2016 | 196 |
| Ovid Medline (R) | Basic Search | Tachysystole | 1996 | 08/12/2016 | 168 |
| CINAHL complete | Boolean/Phrase | Uterine activity | 1976 | 08/12/2016 | 148 |
| CINAHL complete | Boolean/Phrase | Excessive uterine activity | 1976 | 08/12/2016 | 10 |
| CINAHL complete | Boolean/Phrase | XSUA | 1976 | 08/12/2016 | 0 |
| CINAHL complete | Boolean/Phrase | Uterine hyperstimulation | 1976 | 08/12/2016 | 48 |
| CINAHL complete | Boolean/Phrase | Tachysystole | 1976 | 08/12/2016 | 56 |
| Ovid Medline (R) | Basic Search | Uterine activity | 08/12/2016 | 26/09/2019 | 59 |
| Ovid Medline (R) | Basic Search | Excessive uterine activity | 08/12/2016 | 26/09/2019 | 4 |
| Ovid Medline (R) | Basic Search | XSUA | 08/12/2016 | 26/09/2019 | 0 |
| Ovid Medline (R) | Basic Search | Uterine hyperstimulation | 08/12/2016 | 26/09/2019 | 19 |
| Ovid Medline (R) | Basic Search | Tachysystole | 08/12/2016 | 26/09/2019 | 46 |
| ClinicalTrials.gov | Basic Search | Tachysystole | - | 26/09/2019 | 0 |
| ClinicalTrials.gov | Basic Search | Uterine Activity | - | 26/09/2019 | 0 |
| Identified from relevant articles |  |  |  |  | 4 |

## Study Characteristics

Table S2. Characteristics of Included Studies

| Study Name | Study Type | No. Of Labours | Setting | Period | Labour Type | Delivery Type | Method of Uterine Activity Assessment | Period of Labour Assessed | Component of Uterine Activity Assessed | Included Outcomes |
| --- | --- | --- | --- | --- | --- | --- | --- | --- | --- | --- |
| **Heuser et al. 2013** | Retrospective cohort  Groups defined by exposure | 50335  (TS: 11%) | Urban teaching hospital in North America | 2007-2009 | Mixed (spontaneous, induced and augmented) | Mixed  (8% operative vaginal, 12% caesarean) | Un-blinded manual assessment  Tocography type not specified | Recording must have lasted 120 minutes and included delivery | Contraction rate | Apgar score |
| **Stewart et al. 2012** | Prospective Cohort  Groups defined by exposure | 584  (TS: 43%) | Parkland Hospital, Texas, USA. | 2009-2010 | All induced (oral misoprostol) | Mixed  (19% caesarean) | Blinded manual assessment  89% IUPC, 11% external tocography | Four hours from induction | Contraction rate  Contraction duration | Apgar score  Umbilical artery pH  Intubation at delivery |
| **Jonsson et al. 2008** | Case-control | 915  305 cases, 610 controls | Örebro and Uppsala University Hospitals in Sweden | 1994-2004 | Mixed (spontaneous, induced and augmented) | Mixed  (vacuum assisted vaginal [9%], caesarean [8%]) | Blinded manual assessment  Tocography type not specified | Two hours before delivery | Contraction rate | Umbilical artery pH |
| **Mutlu Meydanli et al. 2002** | Prospective Cohort  Groups defined by outcome | 720  (TS: 7%) | Teaching Hospital, Ankara, Turkey | 1999-2000 | All induced (Intravaginal misoprostol) | Mixed  (22% caesarean, 5% operative vaginal) | Blinded manual assessment  External tocography mentioned but internal not explicitly excluded | From Induction to Delivery | Contraction rate | Umbilical artery pH |
| **Ahmed et al. 2016** | Retrospective Cohort  Groups defined by exposure | 8008  (TS: 11%) | Tertiary care facility, New York, USA | 2007-2009 | Spontaneous, unaugmented | Mixed  (normal and assisted vaginal and caesarean) | Concurrent manual assessment with electronic decision support input  Tocography type not specified | Not explicitly reported, likely whole recording | Contraction rate | Apgar score |
| **Mussi et al. 2015** | Prospective Cohort  Groups defined by outcome | 430  (TS: 21%) | San Gerardo Hospital Milan, Italy | 2011 | Induced or augmented only (Oxytocin) | Mixed (11% operative vaginal, 15% caesarean) | Concurrent manual assessment  External tocography | Not explicitly reported, likely whole recording | Combined contraction rate or duration | Umbilical artery pH and base deficit |
| **Smith et al. 2014** | Retrospective Cohort  Groups defined by outcome | 6234  (TS: 18%) | Community teaching hospital, Maryland, USA | 2006-2010 | Mixed  (41% induced, 16% augmented) | Mixed (24% caesarean) | Automated assessment  Tocography type not specified | Up to four hours before delivery | Contraction rate | Umbilical artery base deficit or Apgar score |
| **Bakker at al. 2006** | Retrospective Cohort  Groups defined by outcome | 1433  (TS: not specified) | University Medical Centre, Amsterdam, The Netherlands | 1993-2004 | Mixed  (Oxytocin: 75%, Prostaglandins: 8%) | Vaginal deliveries only | Automated assessment  IUPC | Last hour of first stage, entire second stage | Contraction rate, duration, amplitude, and surface  Relaxation time  Montevideo units  Active planimeter units | Umbilical artery pH |
| **Hamilton et al. 2012** | Case-Control | 3636  (316 cases, 3320 controls) | Urban university teaching hospital in North America | 2004-2010 | Mixed  (41% Induced, 16% augmented) | Mixed  (24% caesarean delivery, 7% operative vaginal delivery) | Automated assessment  Mostly external tocography | Four hours before delivery | Contraction rate | Umbilical artery base deficit |
| **Palanisamy et al. 2019** | Secondary analysis of prospective cohort  Groups defined by exposure | 8,580  (TS^[[1]](#endnote-2)^: 6%) | Single tertiary care institution, St. Louis, Missouri, USA | 2010-2015 | Mixed  (spontaneous, induced and augmented) | Not reported | Blinded manual assessment  Tocography type not specified | One hour before delivery | Contraction rate | Umbilical artery pH  Lactate  Base excess |
| **Bofill et al. 2017** | Secondary analysis of prospective cohort  Groups defined by exposure | 762  (TS: 30%) | University of Mississippi Medical Center | 1999-2004 | Induced labours only (prostaglandins ± oxytocin) | Mixed  (33% caesarean) | Manual assessment of external tocography  Blinding not stated | From induction to delivery | Contraction rate | Apgar score  UA pH  UA BE |
| **Hayes et al. 2013** | Case-control | 726  (237 cases, 489 controls) |  | 2001-2008 | Mixed  (75% induced) |  | As documented by midwife attending labour  External tocography | Whole of monitored labour | Contraction rate | Grade of neonatal encephalopathy |

## Risk of Bias Assessments

Risk of bias assessments were conducted for outcomes included in this review. The hypothesis of this review may not have been the main hypothesis of the included studies. The following assessments are not necessarily a reflection of the overall quality of the included articles.

Table S3. Risk of bias assessment for Heuser et al.

| **SECTION 1: INTERNAL VALIDITY** | **Does this study do it?** |
| --- | --- |
| The study addresses an appropriate and clearly focused question. | Yes ☒ Can’t say □ No □ |
| **SELECTION OF SUBJECTS** | |
| The two groups being studied are selected from source populations that are comparable in all respects other than the factor under investigation. | Yes □ Can’t say □ No ☒^[[2]](#endnote-3)^  Does not apply □ |
| The study indicates how many of the people asked to take part did so, in each of the groups being studied. | Yes ☒^[[3]](#endnote-4)^ No □  Does not apply □ |
| The likelihood that some eligible subjects might have the outcome at the time of enrolment is assessed and taken into account in the analysis. | Yes □ Can’t say □ No □  Does not apply ☒ |
| What percentage of individuals or clusters recruited into each arm of the study dropped out before the study was completed. | Not applicable |
| Comparison is made between full participants and those lost to follow up, by exposure status. | Yes □ Can’t say □ No □  Does not apply ☒ |
| **ASSESSMENT** | |
| The outcomes are clearly defined. | Yes ☒ Can’t say □ No □ |
| The assessment of outcome is made blind to exposure status. If the study is retrospective this may not be applicable. | Yes □ Can’t say □ No ☒^[[4]](#endnote-5)^  Does not apply □ |
| Where blinding was not possible, there is some recognition that knowledge of exposure status could have influenced the assessment of outcome. | Yes □ Can’t say □ No ☒^[[5]](#endnote-6)^ |
| The method of assessment of exposure is reliable. | Yes □ Can’t say ☒^[[6]](#endnote-7)^ No □ |
| Evidence from other sources is used to demonstrate that the method of outcome assessment is valid and reliable. | Yes □ Can’t say □ No ☒  Does not apply □ |
| Exposure level or prognostic factor is assessed more than once. | Yes □ Can’t say □ No ☒  Does not apply □ |
| **CONFOUNDING** | |
| The main potential confounders are identified and taken into account in the design and analysis. | Yes □ Can’t say□ No ☒ |
| **STATISTICAL ANALYSIS** | |
| Have confidence intervals been provided? | Yes □ No ☒^[[7]](#endnote-8)^ |
| **SECTION 2: OVERALL ASSESSMENT OF THE STUDY** | |
| How well was the study done to minimise the risk of bias or confounding?   - Low Risk: Majority of criteria met. Little or no risk of bias. Results unlikely to be changed by further research. - Medium Risk: Most criteria met. Some flaws in the study with an associated risk of bias, Conclusions may change in the light of further studies. - Low quality: Either most criteria not met, or significant flaws relating to key aspects of study design. Conclusions likely to change in the light of further studies. | Low Risk □  Medium Risk ☒  High Risk □  Unclear □ |
| Taking into account clinical considerations, your evaluation of the methodology used, and the statistical power of the study, do you think there is clear evidence of an association between exposure and outcome? | Yes □ Can’t say ☒ No □ |
| Are the results of this study directly applicable to the patient group targeted in this review? | Yes ☒ No □ |
| **SECTION 3: COMMENTS ON STRENGTHS AND LIMITATIONS** | |
| Strengths:   - Large number of participants means study is appropriately powered for outcomes of interest i.e. low Apgar scores   Limitations:   - Main neonatal outcomes were a composite outcome (sepsis, IVH, NEC, pneumothorax or low APGAR score) or NICU admission. These outcome measures were not eligible for inclusion in this review. - Uterine activity was assessed prospectively, so therefore blind to outcome but not blinded to potential confounders. No data on reliability of contraction rate assessment. Were nurses more likely to document tachysystole if other risk factors were present e.g. abnormal foetal heart rate patterns? - Multivariate analysis for potential confounders was presented, but only for caesarean delivery, operative vaginal delivery, NICU admission and composite outcome, and not for included outcome (Apgar score) unfortunately. Apparently multivariate analysis was performed for Apgar score but just reported as showing no significant results. - Multivariate analysis includes appropriate dependant variables, but also variables that are arguably inappropriate because they are plausibly along the casual pathway of the exposure and outcome. For example, oxytocin use was included but plausibly oxytocin use leads to tachysystole and then to low Apgar scores. | |

Table S4. Risk of bias assessment for Stewart et al.

| **SECTION 1: INTERNAL VALIDITY** | **Does this study do it?** |
| --- | --- |
| The study addresses an appropriate and clearly focused question. | Yes ☒ Can’t say □ No □ |
| **SELECTION OF SUBJECTS** | |
| The two groups being studied are selected from source populations that are comparable in all respects other than the factor under investigation. | Yes □ Can’t say ☒^[[8]](#endnote-9)^ No □  Does not apply □ |
| The study indicates how many of the people asked to take part did so, in each of the groups being studied. | Yes ☒^[[9]](#endnote-10)^ No □  Does not apply □ |
| The likelihood that some eligible subjects might have the outcome at the time of enrolment is assessed and taken into account in the analysis. | Yes ☒^[[10]](#endnote-11)^ Can’t say □ No □  Does not apply □ |
| What percentage of individuals or clusters recruited into each arm of the study dropped out before the study was completed. | Not applicable |
| Comparison is made between full participants and those lost to follow up, by exposure status. | Yes □ Can’t say □ No □  Does not apply ☒^[[11]](#endnote-12)^ |
| **ASSESSMENT** | |
| The outcomes are clearly defined. | Yes ☒ Can’t say □ No □ |
| The assessment of outcome is made blind to exposure status. If the study is retrospective this may not be applicable. | Yes □ Can’t say □ No ☒^[[12]](#endnote-13)^  Does not apply □ |
| Where blinding was not possible, there is some recognition that knowledge of exposure status could have influenced the assessment of outcome. | Yes □ Can’t say □ No ☒^[[13]](#endnote-14)^ |
| The method of assessment of exposure is reliable. | Yes □ Can’t say ☒^[[14]](#endnote-15)^ No □ |
| Evidence from other sources is used to demonstrate that the method of outcome assessment is valid and reliable. | Yes □ Can’t say □ No ☒  Does not apply □ |
| Exposure level or prognostic factor is assessed more than once. | Yes □ Can’t say □ No ☒  Does not apply □ |
| **CONFOUNDING** | |
| The main potential confounders are identified and taken into account in the design and analysis. | Yes □ Can’t say□ No ☒ |
| **STATISTICAL ANALYSIS** | |
| Have confidence intervals been provided? | Yes □ No ☒ |
| **SECTION 2: OVERALL ASSESSMENT OF THE STUDY** | |
| How well was the study done to minimise the risk of bias or confounding?   - Low Risk: Majority of criteria met. Little or no risk of bias. Results unlikely to be changed by further research. - Medium Risk: Most criteria met. Some flaws in the study with an associated risk of bias, Conclusions may change in the light of further studies. - Low quality: Either most criteria not met, or significant flaws relating to key aspects of study design. Conclusions likely to change in the light of further studies. | Low Risk □  Medium Risk ☒  High Risk □  Unclear □ |
| Taking into account clinical considerations, your evaluation of the methodology used, and the statistical power of the study, do you think there is clear evidence of an association between exposure and outcome? | Yes □ Can’t say ☒ No □ |
| Are the results of this study directly applicable to the patient group targeted in this review? | Yes ☒ No □ |
| **SECTION 3: COMMENTS ON STRENGTHS AND LIMITATIONS** | |
| Strengths:   - All eligible women were included. Study likely had low risk of selection bias. - Standard practice for management of tachysystole was stated. (Adherence to standard practice not reported however.) - One of the few studies to look at early labour specifically - Associated foetal heart rate analysis   Limitations:   - No data on exclusions - Foetal vulnerability score was primary outcome, but not included in this review as contains NICU admission as a component and data on separate outcomes was available. - Main limitation is study power. Small numbers of babies had the adverse outcomes of relevance to this review. - Authors report on stillbirth as an outcome, but foetal demise is an exclusion criterion. Ideally, cases of foetal demise would be eligible for inclusion and reported as an outcome. | |

Table S5. Risk of bias assessment for Jonsson et al.

| **SECTION 1: INTERNAL VALIDITY** | **Does this study do it?** |
| --- | --- |
| The study addresses an appropriate and clearly focused question. | Yes ☒ Can’t say □ No □ |
| **SELECTION OF SUBJECTS** | |
| The cases and controls are taken from comparable populations. | Yes ☒^[[15]](#endnote-16)^ Can’t say □ No □ |
| The same exclusion criteria are used for both cases and controls. | Yes ☒ Can’t say □ No □ |
| What percentage of each group (cases and controls) participated in the study? | Cases:100% (72% with trace)  Controls: 100% (62.4% with trace) |
| Comparison is made between participants and non-participants to establish their similarities or differences. | Yes ☒^[[16]](#endnote-17)^ Can’t say □ No □ |
| Cases are clearly defined and differentiated from controls. | Yes ☒ Can’t say □ No □ |
| It is clearly established that controls are non-cases. | Yes ☒ Can’t say □ No □ |
| **ASSESSMENT** | |
| Measures will have been taken to prevent knowledge of primary exposure influencing case ascertainment. | Yes ☒^[[17]](#endnote-18)^ Can’t say □ No □ |
| Exposure status is measured in a standard, valid and reliable way. | Yes □ Can’t say ☒^[[18]](#endnote-19)^ No |
| **CONFOUNDING** | |
| The main potential confounders are identified and taken into account in the design and analysis. | Yes □ Can’t say□ No ☒^[[19]](#endnote-20)^ |
| **STATISTICAL ANALYSIS** | |
| Have confidence intervals been provided? | Yes ☒^[[20]](#endnote-21)^ No ☒ |
| **SECTION 2: OVERALL ASSESSMENT OF THE STUDY** | |
| How well was the study done to minimise the risk of bias or confounding?   - Low Risk: Majority of criteria met. Little or no risk of bias. Results unlikely to be changed by further research. - Medium Risk: Most criteria met. Some flaws in the study with an associated risk of bias, Conclusions may change in the light of further studies. - High Risk: Either most criteria not met, or significant flaws relating to key aspects of study design. Conclusions likely to change in the light of further studies. | Low Risk □  Medium Risk ☒  High Risk □  Unclear □ |
| Taking into account clinical considerations, your evaluation of the methodology used, and the statistical power of the study, do you think there is clear evidence of an association between exposure and outcome? | Yes □ Can’t say ☒ No □ |
| Are the results of this study directly applicable to the patient group targeted in this review? | Yes ☒ No □ |
| **SECTION 3: COMMENTS ON STRENGTHS AND LIMITATIONS** | |
| Strengths:   - Objective outcome measure - Blinded assessment of traces. - Appropriate sample size for outcome given case-control study design   Limitations:   - Controls were matched for parity. If tachysystole is due to lower parity, then this is not a confounder and should not be controlled for. - Cases from 1994 to 2004. Possibly significant changes in obstetric practices since start of this study. - Rate of missing (16%) or inadequate traces is high Only 66.6% of subjects had interpretable traces. Most inadequate traces were from high pH group. Possibly leading to an underestimate of effect. - Only one vessel sampled for majority of subjects. Could umbilical vein have been sampled in some? - Multivariate analysis controlled for maternal age and gestation only, not for e.g. duration of pushing or malpresentation both of which were associated with adverse outcomes in this study. | |

Table S6. Risk of bias assessment for Mutlu Meydanli et al.

| **SECTION 1: INTERNAL VALIDITY** | **Does this study do it?** |
| --- | --- |
| The study addresses an appropriate and clearly focused question. | Yes ☒ Can’t say □ No □ |
| **SELECTION OF SUBJECTS** | |
| The two groups being studied are selected from source populations that are comparable in all respects other than the factor under investigation. | Yes □ Can’t say ☒^[[21]](#endnote-22)^ No □  Does not apply □ |
| The study indicates how many of the people asked to take part did so, in each of the groups being studied. | Yes ☒^[[22]](#endnote-23)^ No □  Does not apply □ |
| The likelihood that some eligible subjects might have the outcome at the time of enrolment is assessed and taken into account in the analysis. | Yes □ Can’t say □ No □  Does not apply ☒ |
| What percentage of individuals or clusters recruited into each arm of the study dropped out before the study was completed. | Not applicable |
| Comparison is made between full participants and those lost to follow up, by exposure status. | Yes □ Can’t say □ No □  Does not apply ☒ |
| **ASSESSMENT** | |
| The outcomes are clearly defined. | Yes ☒ Can’t say □ No □ |
| The assessment of outcome is made blind to exposure status. If the study is retrospective this may not be applicable. | Yes □ Can’t say □ No  Does not apply ☒ |
| Where blinding was not possible, there is some recognition that knowledge of exposure status could have influenced the assessment of outcome. | Yes □ Can’t say □ No □ Does not apply ☒ |
| The method of assessment of exposure is reliable. | Yes □ Can’t say ☒^[[23]](#endnote-24)^ No □ |
| Evidence from other sources is used to demonstrate that the method of outcome assessment is valid and reliable. | Yes □ Can’t say □ No ☒  Does not apply □ |
| Exposure level or prognostic factor is assessed more than once. | Yes □ Can’t say □ No ☒  Does not apply □ |
| **CONFOUNDING** | |
| The main potential confounders are identified and taken into account in the design and analysis. | Yes □ Can’t say□ No ☒^[[24]](#endnote-25)^ |
| **STATISTICAL ANALYSIS** | |
| Have confidence intervals been provided? | Yes ☒^[[25]](#endnote-26)^ No ☒ |
| **SECTION 2: OVERALL ASSESSMENT OF THE STUDY** | |
| How well was the study done to minimise the risk of bias or confounding?   - Low Risk: Majority of criteria met. Little or no risk of bias. Results unlikely to be changed by further research. - Medium Risk: Most criteria met. Some flaws in the study with an associated risk of bias, Conclusions may change in the light of further studies. - High Risk: Either most criteria not met, or significant flaws relating to key aspects of study design. Conclusions likely to change in the light of further studies. | Low Risk □  Medium Risk ☒  High Risk □  Unclear □ |
| Taking into account clinical considerations, your evaluation of the methodology used, and the statistical power of the study, do you think there is clear evidence of an association between exposure and outcome? | Yes □ Can’t say ☒ No □ |
| Are the results of this study directly applicable to the patient group targeted in this review? | Yes ☒^[[26]](#endnote-27)^ No □ |
| **SECTION 3: COMMENTS ON STRENGTHS AND LIMITATIONS** | |
| Strengths:   - Prospective - Objective outcome measure - Blinded assessment of full length of traces   Limitations:   - Main outcome was a composite outcome (neonatal death, UA pH ≤7.15, or caesarean delivery non-reassuring FHR tracings). Delivery method was not eligible for inclusion in this study and so the primary outcome was not included. - Relative risk, but not group numbers were reported for the eligible outcome (pH). - Cases from 1999 to 2000. Possibly significant changes in obstetric practices since start of this study. - All cases had a Bishop’s score of ≤4. The generalisability of results beyond this group is questionable. - A logistic regression odds ratio was reported for tachysystole and the risk of acidemia, but no details of model supplied. Impossible to know if confounders accounted for. - Statistically significant result but wide relative risk confidence interval for outcome included in this study. - Low rate of tachysystole raises further questions about generalisability. | |

Table S7. Risk of bias assessment for Ahmed et al.

| **SECTION 1: INTERNAL VALIDITY** | **Does this study do it?** |
| --- | --- |
| The study addresses an appropriate and clearly focused question. | Yes ☒ Can’t say □ No □ |
| **SELECTION OF SUBJECTS** | |
| The two groups being studied are selected from source populations that are comparable in all respects other than the factor under investigation. | Yes □ Can’t say ☒^[[27]](#endnote-28)^ No □  Does not apply □ |
| The study indicates how many of the people asked to take part did so, in each of the groups being studied. | Yes ☒^[[28]](#endnote-29)^ No □  Does not apply □ |
| The likelihood that some eligible subjects might have the outcome at the time of enrolment is assessed and taken into account in the analysis. | Yes □ Can’t say □ No □  Does not apply ☒ |
| What percentage of individuals or clusters recruited into each arm of the study dropped out before the study was completed. | Not applicable |
| Comparison is made between full participants and those lost to follow up, by exposure status. | Yes □ Can’t say □ No □  Does not apply ☒ |
| **ASSESSMENT** | |
| The outcomes are clearly defined. | Yes ☒ Can’t say □ No □ |
| The assessment of outcome is made blind to exposure status. If the study is retrospective this may not be applicable. | Yes □ Can’t say □ No  Does not apply ☒ |
| Where blinding was not possible, there is some recognition that knowledge of exposure status could have influenced the assessment of outcome. | Yes □ Can’t say □ No □ Does not apply ☒ |
| The method of assessment of exposure is reliable. | Yes □ Can’t say ☒^[[29]](#endnote-30)^ No □ |
| Evidence from other sources is used to demonstrate that the method of outcome assessment is valid and reliable. | Yes □ Can’t say □ No ☒  Does not apply □ |
| Exposure level or prognostic factor is assessed more than once. | Yes □ Can’t say □ No ☒  Does not apply □ |
| **CONFOUNDING** | |
| The main potential confounders are identified and taken into account in the design and analysis. | Yes □ Can’t say□ No ☒^[[30]](#endnote-31)^ |
| **STATISTICAL ANALYSIS** | |
| Have confidence intervals been provided? | Yes ☒ No ☒ |
| **SECTION 2: OVERALL ASSESSMENT OF THE STUDY** | |
| How well was the study done to minimise the risk of bias or confounding?   - Low Risk: Majority of criteria met. Little or no risk of bias. Results unlikely to be changed by further research. - Medium Risk: Most criteria met. Some flaws in the study with an associated risk of bias, Conclusions may change in the light of further studies. - High Risk: Either most criteria not met, or significant flaws relating to key aspects of study design. Conclusions likely to change in the light of further studies. | Low Risk □  Medium Risk ☒  High Risk □  Unclear □ |
| Taking into account clinical considerations, your evaluation of the methodology used, and the statistical power of the study, do you think there is clear evidence of an association between exposure and outcome? | Yes □ Can’t say ☒ No □ |
| Are the results of this study directly applicable to the patient group targeted in this review? | Yes ☒^[[31]](#endnote-32)^ No □ |
| **SECTION 3: COMMENTS ON STRENGTHS AND LIMITATIONS** | |
| Strengths:   - Groups divided by uterine activity and many characteristics of each group described. - Relevant and widely used outcome measure i.e. - Also incorporates foetal heart rate analysis and caesarean delivery for NRFHR traces as an independent variable.   Limitations:   - Included NICU admission as one of four major neonatal outcomes. This outcome was considered too heterogenous to be included as an outcome for the purposes of this review. - All subjects had spontaneous unaugmented labour. The generalisability of results beyond this group is questionable. - Although baseline characteristics influencing the risk of tachysystole were reported, no adjusted likelihood values for neonatal outcomes were reported. - Very low rate of low Apgar scores means that study was underpowered for detecting changes in this outcome in this population. | |

Table S8. Risk of bias assessment for Mussi et al.

| **SECTION 1: INTERNAL VALIDITY** | **Does this study do it?** |
| --- | --- |
| The study addresses an appropriate and clearly focused question. | Yes ☒ Can’t say □ No □ |
| **SELECTION OF SUBJECTS** | |
| The two groups being studied are selected from source populations that are comparable in all respects other than the factor under investigation. | Yes □ Can’t say ☒^[[32]](#endnote-33)^ No □  Does not apply □ |
| The study indicates how many of the people asked to take part did so, in each of the groups being studied. | Yes ☒^[[33]](#endnote-34)^ No □  Does not apply □ |
| The likelihood that some eligible subjects might have the outcome at the time of enrolment is assessed and taken into account in the analysis. | Yes □ Can’t say □ No □  Does not apply ☒ |
| What percentage of individuals or clusters recruited into each arm of the study dropped out before the study was completed. | Not applicable |
| Comparison is made between full participants and those lost to follow up, by exposure status. | Yes □ Can’t say □ No □  Does not apply ☒ |
| **ASSESSMENT** | |
| The outcomes are clearly defined. | Yes ☒ Can’t say □ No □ |
| The assessment of outcome is made blind to exposure status. If the study is retrospective this may not be applicable. | Yes ☒ Can’t say □ No  Does not apply □ |
| Where blinding was not possible, there is some recognition that knowledge of exposure status could have influenced the assessment of outcome. | Yes □ Can’t say □ No □ Does not apply ☒ |
| The method of assessment of exposure is reliable. | Yes □ Can’t say ☒^[[34]](#endnote-35)^ No □ |
| Evidence from other sources is used to demonstrate that the method of outcome assessment is valid and reliable. | Yes □ Can’t say □ No ☒  Does not apply □ |
| Exposure level or prognostic factor is assessed more than once. | Yes □ Can’t say □ No ☒  Does not apply □ |
| **CONFOUNDING** | |
| The main potential confounders are identified and taken into account in the design and analysis. | Yes □ Can’t say□ No ☒^[[35]](#endnote-36)^ |
| **STATISTICAL ANALYSIS** | |
| Have confidence intervals been provided? | Yes □ No ☒ |
| **SECTION 2: OVERALL ASSESSMENT OF THE STUDY** | |
| How well was the study done to minimise the risk of bias or confounding?   - Low Risk: Majority of criteria met. Little or no risk of bias. Results unlikely to be changed by further research. - Medium Risk: Most criteria met. Some flaws in the study with an associated risk of bias, Conclusions may change in the light of further studies. - High Risk: Either most criteria not met, or significant flaws relating to key aspects of study design. Conclusions likely to change in the light of further studies. | Low Risk □  Medium Risk □  High Risk ☒  Unclear □ |
| Taking into account clinical considerations, your evaluation of the methodology used, and the statistical power of the study, do you think there is clear evidence of an association between exposure and outcome? | Yes □ Can’t say □ No ☒ |
| Are the results of this study directly applicable to the patient group targeted in this review? | Yes ☒^[[36]](#endnote-37)^ No □ |
| **SECTION 3: COMMENTS ON STRENGTHS AND LIMITATIONS** | |
| Strengths:   - Explicitly stated oxytocin management protocol. This makes clear to which clinical settings the results may be generalisable.   Limitations:   - Small number of cases means that study was underpowered for detecting increased rates of significant acidaemia. - Groups divided by acidaemia rather than tachysystole for most descriptors. - Uterine tachysystole and hypertonus treated as equivalent exposures, but supporting data provided. The relative contribution of each component was not detailed. - Implicit hypothesis of major statistical test in this article is that the presence of tachysystole is linearly correlated with pH or BE. In contrast, the hypothesis of this review for this particular outcome is that tachysystole is associated with an increased likelihood of acidaemia. - Confidence intervals for primary outcomes (Pearson correlation) are not provided. - All subjects received oxytocin during labour. The generalisability of results beyond this group is questionable. | |

Table S9. Risk of bias assessment for Smith et al.

| **SECTION 1: INTERNAL VALIDITY** | **Does this study do it?** |
| --- | --- |
| The study addresses an appropriate and clearly focused question. | Yes ☒ Can’t say □ No □ |
| **SELECTION OF SUBJECTS** | |
| The two groups being studied are selected from source populations that are comparable in all respects other than the factor under investigation. | Yes □ Can’t say ☒^[[37]](#endnote-38)^ No □  Does not apply □ |
| The study indicates how many of the people asked to take part did so, in each of the groups being studied. | Yes ☒^[[38]](#endnote-39)^ No □  Does not apply □ |
| The likelihood that some eligible subjects might have the outcome at the time of enrolment is assessed and taken into account in the analysis. | Yes □ Can’t say □ No □  Does not apply ☒ |
| What percentage of individuals or clusters recruited into each arm of the study dropped out before the study was completed. | Not applicable |
| Comparison is made between full participants and those lost to follow up, by exposure status. | Yes □ Can’t say □ No □  Does not apply ☒ |
| **ASSESSMENT** | |
| The outcomes are clearly defined. | Yes ☒ Can’t say □ No □ |
| The assessment of outcome is made blind to exposure status. If the study is retrospective this may not be applicable. | Yes □ Can’t say ☒^[[39]](#endnote-40)^ No  Does not apply □ |
| Where blinding was not possible, there is some recognition that knowledge of exposure status could have influenced the assessment of outcome. | Yes □ Can’t say □ No □ Does not apply ☒ |
| The method of assessment of exposure is reliable. | Yes □ Can’t say ☒^[[40]](#endnote-41)^ No □ |
| Evidence from other sources is used to demonstrate that the method of outcome assessment is valid and reliable. | Yes □ Can’t say □ No ☒  Does not apply □ |
| Exposure level or prognostic factor is assessed more than once. | Yes □ Can’t say □ No ☒  Does not apply □ |
| **CONFOUNDING** | |
| The main potential confounders are identified and taken into account in the design and analysis. | Yes □ Can’t say□ No ☒^[[41]](#endnote-42)^ |
| **STATISTICAL ANALYSIS** | |
| Have confidence intervals been provided? | Yes □ No ☒ |
| **SECTION 2: OVERALL ASSESSMENT OF THE STUDY** | |
| How well was the study done to minimise the risk of bias or confounding?   - Low Risk: Majority of criteria met. Little or no risk of bias. Results unlikely to be changed by further research. - Medium Risk: Most criteria met. Some flaws in the study with an associated risk of bias, Conclusions may change in the light of further studies. - High Risk: Either most criteria not met, or significant flaws relating to key aspects of study design. Conclusions likely to change in the light of further studies. | Low Risk ☒  Medium Risk □  High Risk □  Unclear □ |
| Taking into account clinical considerations, your evaluation of the methodology used, and the statistical power of the study, do you think there is clear evidence of an association between exposure and outcome? | Yes □ Can’t say □No ☒ |
| Are the results of this study directly applicable to the patient group targeted in this review? | Yes ☒ No □ |
| **SECTION 3: COMMENTS ON STRENGTHS AND LIMITATIONS** | |
| Overall, a relatively high-quality study.  Strengths:   - Specific oxytocin management protocol - Groups divided by uterine activity and many characteristics of each group described. - Adequate sample size - Also incorporates foetal heart rate analysis and caesarean delivery for NRFHR traces as an independent variable.   Limitations:   - No breakdown of composite outcome into individual components - Not clear how many subjects had cord blood analysis - Automated assessment of uterine activity but no data on reliability of algorithm referenced or provided. (Data referenced on reliability of deceleration identification, but no data for uterine activity.) - Potential conflict of interest: three of five authors were employed by company which produced uterine activity analysis software. - No adjustment for confounding. (Result not significant, so this comment less relevant.) | |

Table S10. Risk of bias assessment for Bakker et al.

| **SECTION 1: INTERNAL VALIDITY** | **Does this study do it?** |
| --- | --- |
| The study addresses an appropriate and clearly focused question. | Yes ☒ Can’t say □ No □ |
| **SELECTION OF SUBJECTS** | |
| The two groups being studied are selected from source populations that are comparable in all respects other than the factor under investigation. | Yes □ Can’t say ☒^[[42]](#endnote-43)^ No □  Does not apply □ |
| The study indicates how many of the people asked to take part did so, in each of the groups being studied. | Yes ☒^[[43]](#endnote-44)^ No □  Does not apply □ |
| The likelihood that some eligible subjects might have the outcome at the time of enrolment is assessed and taken into account in the analysis. | Yes □ Can’t say □ No □  Does not apply ☒ |
| What percentage of individuals or clusters recruited into each arm of the study dropped out before the study was completed. | Not applicable |
| Comparison is made between full participants and those lost to follow up, by exposure status. | Yes □ Can’t say □ No □  Does not apply ☒ |
| **ASSESSMENT** | |
| The outcomes are clearly defined. | Yes ☒ Can’t say □ No □ |
| The assessment of outcome is made blind to exposure status. If the study is retrospective this may not be applicable. | Yes ☒ Can’t say □ No  Does not apply □ |
| Where blinding was not possible, there is some recognition that knowledge of exposure status could have influenced the assessment of outcome. | Yes □ Can’t say □ No □ Does not apply ☒ |
| The method of assessment of exposure is reliable. | Yes □ Can’t say ☒^[[44]](#endnote-45)^ No □ |
| Evidence from other sources is used to demonstrate that the method of outcome assessment is valid and reliable. | Yes □ Can’t say □ No ☒  Does not apply □ |
| Exposure level or prognostic factor is assessed more than once. | Yes □ Can’t say □ No ☒  Does not apply □ |
| **CONFOUNDING** | |
| The main potential confounders are identified and taken into account in the design and analysis. | Yes □ Can’t say□ No ☒^[[45]](#endnote-46)^ |
| **STATISTICAL ANALYSIS** | |
| Have confidence intervals been provided? | Yes □ No ☒ |
| **SECTION 2: OVERALL ASSESSMENT OF THE STUDY** | |
| How well was the study done to minimise the risk of bias or confounding?   - Low Risk: Majority of criteria met. Little or no risk of bias. Results unlikely to be changed by further research. - Medium Risk: Most criteria met. Some flaws in the study with an associated risk of bias, Conclusions may change in the light of further studies. - High Risk: Either most criteria not met, or significant flaws relating to key aspects of study design. Conclusions likely to change in the light of further studies. | Low Risk ☒  Medium Risk □  High Risk □  Unclear □ |
| Taking into account clinical considerations, your evaluation of the methodology used, and the statistical power of the study, do you think there is clear evidence of an association between exposure and outcome? | Yes ☒ Can’t say □ No □ |
| Are the results of this study directly applicable to the patient group targeted in this review? | Yes ☒^[[46]](#endnote-47)^ No □ |
| **SECTION 3: COMMENTS ON STRENGTHS AND LIMITATIONS** | |
| Overall, a relatively high-quality study, but some key limitations present.  Strengths:   - Use of IUPC recordings only and detailed analysis of uterine activity. (This is also a limitation as the results may not be generalisable to external recordings.) - Robust umbilical cord blood analysis with exclusion where sampling of umbilical vein could not be ruled out. - Appropriate sample size   Limitations:   - Cohort all had an indication for IUPC monitoring and so may not be reflective of the general population i.e. cohort was mainly - No multivariate analysis to account for possible confounders e.g. chorioamnionitis - Labours took place from 1993 to 2004. Changes in obstetric practice since then may invalidate the results. - Contraction rate only reported as a mean value per labour rather than a dichotomous or categorised variable. Not a limitation per se, but the lack of reference to tachysystole limits comparison with other studies. - Groups divided by acidaemia rather than tachysystole for most descriptors. - Automated analysis of IUPC recordings means assessment was definitely blinded to outcomes but no data presented on the reliability of the algorithm. | |

Table S11. Risk of bias assessment for Hamilton et al.

| **SECTION 1: INTERNAL VALIDITY** | **Does this study do it?** |
| --- | --- |
| The study addresses an appropriate and clearly focused question. | Yes ☒ Can’t say □ No □ |
| **SELECTION OF SUBJECTS** | |
| The two groups being studied are selected from source populations that are comparable in all respects other than the factor under investigation. | Yes □ Can’t say ☒^[[47]](#endnote-48)^ No □  Does not apply □ |
| The study indicates how many of the people asked to take part did so, in each of the groups being studied. | Yes ☒^[[48]](#endnote-49)^ No □  Does not apply □ |
| The likelihood that some eligible subjects might have the outcome at the time of enrolment is assessed and taken into account in the analysis. | Yes □ Can’t say □ No □  Does not apply ☒ |
| What percentage of individuals or clusters recruited into each arm of the study dropped out before the study was completed. | Not applicable |
| Comparison is made between full participants and those lost to follow up, by exposure status. | Yes □ Can’t say □ No □  Does not apply ☒ |
| **ASSESSMENT** | |
| The outcomes are clearly defined. | Yes ☒ Can’t say □ No □ |
| The assessment of outcome is made blind to exposure status. If the study is retrospective this may not be applicable. | Yes ☒ Can’t say □ No  Does not apply □ |
| Where blinding was not possible, there is some recognition that knowledge of exposure status could have influenced the assessment of outcome. | Yes □ Can’t say □ No □ Does not apply ☒ |
| The method of assessment of exposure is reliable. | Yes □ Can’t say ☒^[[49]](#endnote-50)^ No □ |
| Evidence from other sources is used to demonstrate that the method of outcome assessment is valid and reliable. | Yes □ Can’t say □ No ☒  Does not apply □ |
| Exposure level or prognostic factor is assessed more than once. | Yes □ Can’t say □ No ☒  Does not apply □ |
| **CONFOUNDING** | |
| The main potential confounders are identified and taken into account in the design and analysis. | Yes □ Can’t say□ No ☒^[[50]](#endnote-51)^ |
| **STATISTICAL ANALYSIS** | |
| Have confidence intervals been provided? | Yes □ No ☒ |
| **SECTION 2: OVERALL ASSESSMENT OF THE STUDY** | |
| How well was the study done to minimise the risk of bias or confounding?   - Low Risk: Majority of criteria met. Little or no risk of bias. Results unlikely to be changed by further research. - Medium Risk: Most criteria met. Some flaws in the study with an associated risk of bias, Conclusions may change in the light of further studies. - High Risk: Either most criteria not met, or significant flaws relating to key aspects of study design. Conclusions likely to change in the light of further studies. | Low Risk  Medium Risk □  High Risk ☒  Unclear □ |
| Taking into account clinical considerations, your evaluation of the methodology used, and the statistical power of the study, do you think there is clear evidence of an association between exposure and outcome? | Yes ☒ Can’t say □ No □ |
| Are the results of this study directly applicable to the patient group targeted in this review? | Yes ☒^[[51]](#endnote-52)^ No □ |
| **SECTION 3: COMMENTS ON STRENGTHS AND LIMITATIONS** | |
| Strengths:   - Sophisticated automated detection of uterine activity allows analysis of trends over time. - Appropriate sample size - Routine blood gas sampling in this cohort - Detailed associated foetal heart rate assessment   Limitations:   - Conflict of interest. Several authors employed by manufacturers of software used for interpretation. - Babies with metabolic acidaemia together with signs of encephalopathy were excluded. - No data in dataset to facilitate analysis of potential confounders and multivariate analysis - Automated analysis of recordings means assessment was definitely blinded to outcomes but no data presented on the reliability of the algorithm. | |

Table S12. Risk of bias assessment for Palanisamy et al.

| **SECTION 1: INTERNAL VALIDITY** | **Does this study do it?** |
| --- | --- |
| The study addresses an appropriate and clearly focused question. | Yes ☒ Can’t say □ No □ |
| **SELECTION OF SUBJECTS** | |
| The two groups being studied are selected from source populations that are comparable in all respects other than the factor under investigation. | Yes □ Can’t say ☒^[[52]](#endnote-53)^ No □  Does not apply □ |
| The study indicates how many of the people asked to take part did so, in each of the groups being studied. | Yes ☒^[[53]](#endnote-54)^ No □  Does not apply □ |
| The likelihood that some eligible subjects might have the outcome at the time of enrolment is assessed and taken into account in the analysis. | Yes □ Can’t say □ No □  Does not apply ☒ |
| What percentage of individuals or clusters recruited into each arm of the study dropped out before the study was completed. | Not applicable |
| Comparison is made between full participants and those lost to follow up, by exposure status. | Yes □ Can’t say □ No □  Does not apply ☒ |
| **ASSESSMENT** | |
| The outcomes are clearly defined. | Yes ☒ Can’t say □ No □ |
| The assessment of outcome is made blind to exposure status. If the study is retrospective this may not be applicable. | Yes ☒ Can’t say □ No  Does not apply □ |
| Where blinding was not possible, there is some recognition that knowledge of exposure status could have influenced the assessment of outcome. | Yes □ Can’t say □ No □ Does not apply ☒ |
| The method of assessment of exposure is reliable. | Yes □ Can’t say ☒^[[54]](#endnote-55)^ No □ |
| Evidence from other sources is used to demonstrate that the method of outcome assessment is valid and reliable. | Yes □ Can’t say □ No ☒  Does not apply □ |
| Exposure level or prognostic factor is assessed more than once. | Yes □ Can’t say □ No ☒  Does not apply □ |
| **CONFOUNDING** | |
| The main potential confounders are identified and taken into account in the design and analysis. | Yes □ Can’t say□ No ☒^[[55]](#endnote-56)^ |
| **STATISTICAL ANALYSIS** | |
| Have confidence intervals been provided? | Yes ☒ No □ |
| **SECTION 2: OVERALL ASSESSMENT OF THE STUDY** | |
| How well was the study done to minimise the risk of bias or confounding?   - Low Risk: Majority of criteria met. Little or no risk of bias. Results unlikely to be changed by further research. - Medium Risk: Most criteria met. Some flaws in the study with an associated risk of bias, Conclusions may change in the light of further studies. - High Risk: Either most criteria not met, or significant flaws relating to key aspects of study design. Conclusions likely to change in the light of further studies. | Low Risk ☒  Medium Risk □  High Risk □  Unclear □ |
| Taking into account clinical considerations, your evaluation of the methodology used, and the statistical power of the study, do you think there is clear evidence of an association between exposure and outcome? | Yes ☒ Can’t say □ No □ |
| Are the results of this study directly applicable to the patient group targeted in this review? | Yes ☒ No □ |
| **SECTION 3: COMMENTS ON STRENGTHS AND LIMITATIONS** | |
| Definition of tachysystole for main analysis was over one hour. Therefore, definition of TS is slightly different than the ACOG standard. TS result for each half hour period in last hour before delivery were reported but for TS is either period which would be the standard definition used in most other studies. Total TS numbers for each half hour period i.e denominators for analysis were not reported in article but provided by authors on request:  “Final 31-60 denominator is for Always Tachy 1,132 and Never Tachy 7,448. However, there could have been some missing (n= 114 and 108) for lactate and excess, respectively. Last 30 denominator is for Always Tachy 1,247 and Never Tachy 7,333. Same missing applies here.”  Strengths:   - Universal cord gas policy (Although, no data on steps to exclude those with sampling errors.) - Blinded analysis of uterine activity by trained obstetric nurses. - Appropriate sample size   Limitations:   - No data on mode of delivery. - No data on reliability of uterine activity analysis. - Limited multivariate analysis which included maternal age and BMI only, but not pyrexia or other signs of chorioamnionitis. - Included NICU admission as outcome, but no breakdown according to reason for admission. | |

Table S13. Risk of bias assessment for Bofill et al.

| **SECTION 1: INTERNAL VALIDITY** | **Does this study do it?** |
| --- | --- |
| The study addresses an appropriate and clearly focused question. | Yes ☒ Can’t say □ No □ |
| **SELECTION OF SUBJECTS** | |
| The two groups being studied are selected from source populations that are comparable in all respects other than the factor under investigation. | Yes □ Can’t say □ No ☒^[[56]](#endnote-57)^  Does not apply □ |
| The study indicates how many of the people asked to take part did so, in each of the groups being studied. | Yes □ No ☒^[[57]](#endnote-58)^  Does not apply □ |
| The likelihood that some eligible subjects might have the outcome at the time of enrolment is assessed and taken into account in the analysis. | Yes □ Can’t say □ No □  Does not apply ☒ |
| What percentage of individuals or clusters recruited into each arm of the study dropped out before the study was completed. | Not applicable |
| Comparison is made between full participants and those lost to follow up, by exposure status. | Yes □ Can’t say □ No □  Does not apply ☒ |
| **ASSESSMENT** | |
| The outcomes are clearly defined. | Yes ☒ Can’t say □ No □ |
| The assessment of outcome is made blind to exposure status. If the study is retrospective this may not be applicable. | Yes ☒^[[58]](#endnote-59)^ Can’t say □ No  Does not apply □ |
| Where blinding was not possible, there is some recognition that knowledge of exposure status could have influenced the assessment of outcome. | Yes □ Can’t say □ No □ Does not apply ☒ |
| The method of assessment of exposure is reliable. | Yes □ Can’t say ☒^[[59]](#endnote-60)^ No □ |
| Evidence from other sources is used to demonstrate that the method of outcome assessment is valid and reliable. | Yes □ Can’t say □ No ☒  Does not apply □ |
| Exposure level or prognostic factor is assessed more than once. | Yes □ Can’t say □ No ☒  Does not apply □ |
| **CONFOUNDING** | |
| The main potential confounders are identified and taken into account in the design and analysis. | Yes □ Can’t say□ No ☒^[[60]](#endnote-61)^ |
| **STATISTICAL ANALYSIS** | |
| Have confidence intervals been provided? | Yes □ No ☒ |
| **SECTION 2: OVERALL ASSESSMENT OF THE STUDY** | |
| How well was the study done to minimise the risk of bias or confounding?   - Low Risk: Majority of criteria met. Little or no risk of bias. Results unlikely to be changed by further research. - Medium Risk: Most criteria met. Some flaws in the study with an associated risk of bias, Conclusions may change in the light of further studies. - High Risk: Either most criteria not met, or significant flaws relating to key aspects of study design. Conclusions likely to change in the light of further studies. | Low Risk □  Medium Risk □  High Risk ☒  Unclear □ |
| Taking into account clinical considerations, your evaluation of the methodology used, and the statistical power of the study, do you think there is clear evidence of an association between exposure and outcome? | Yes □ Can’t say ☒ No □ |
| Are the results of this study directly applicable to the patient group targeted in this review? | Yes ☒ No □ |
| **SECTION 3: COMMENTS ON STRENGTHS AND LIMITATIONS** | |
| Strengths:   - Data provided on the mode of delivery and the indication for caesarean delivery according to tachysystole status   Limitations:   - Subject had unfavourable Bishop’s score and underwent cervical ripening. Generalisability of results beyond this group is questionable. - Small sample size - Outcomes for subjects with 1-2 episodes of tachysystole were not described - Uterine activity assessment was not reported to be blind to clinical details. - Number with cord pH testing not specified - No multivariate analysis performed - Data is from 1999 – 2004. | |

Table S14. Risk of bias assessment for Hayes et al.

| **SECTION 1: INTERNAL VALIDITY** | **Does this study do it?** |
| --- | --- |
| The study addresses an appropriate and clearly focused question. | Yes ☒ Can’t say □ No □ |
| **SELECTION OF SUBJECTS** | |
| The cases and controls are taken from comparable populations. | Yes ☒^[[61]](#endnote-62)^ Can’t say □ No □ |
| The same exclusion criteria are used for both cases and controls. | Yes ☒ Can’t say □ No □ |
| What percentage of each group (cases and controls) participated in the study? | Cases: 97% (237/245) + 3% no electronic monitoring (n=7) + 8% intermittent monitoring (n=19)  Controls: 100% (489/490) + 10% no electronic monitoring (n=50) + 17% intermittent monitoring (n=84) |
| Comparison is made between participants and non-participants to establish their similarities or differences. | Yes ☒^[[62]](#endnote-63)^ Can’t say □ No □ |
| Cases are clearly defined and differentiated from controls. | Yes ☒ Can’t say □ No □ |
| It is clearly established that controls are non-cases. | Yes ☒ Can’t say □ No □ |
| **ASSESSMENT** | |
| Measures will have been taken to prevent knowledge of primary exposure influencing case ascertainment. | Yes □ Can’t say □ No ☒^[[63]](#endnote-64)^ |
| Exposure status is measured in a standard, valid and reliable way. | Yes □ Can’t say ☒^[[64]](#endnote-65)^ No |
| **CONFOUNDING** | |
| The main potential confounders are identified and taken into account in the design and analysis. | Yes ☒^[[65]](#endnote-66)^ Can’t say □ No □ |
| **STATISTICAL ANALYSIS** | |
| Have confidence intervals been provided? | Yes ☒ No □ |
| **SECTION 2: OVERALL ASSESSMENT OF THE STUDY** | |
| How well was the study done to minimise the risk of bias or confounding?   - Low Risk: Majority of criteria met. Little or no risk of bias. Results unlikely to be changed by further research. - Medium Risk: Most criteria met. Some flaws in the study with an associated risk of bias, Conclusions may change in the light of further studies. - High Risk: Either most criteria not met, or significant flaws relating to key aspects of study design. Conclusions likely to change in the light of further studies. | Low Risk □  Medium Risk ☒^[[66]](#endnote-67)^  High Risk □  Unclear □ |
| Taking into account clinical considerations, your evaluation of the methodology used, and the statistical power of the study, do you think there is clear evidence of an association between exposure and outcome? | Yes □ Can’t say ☒ No □ |
| Are the results of this study directly applicable to the patient group targeted in this review? | Yes ☒ No □ |
| **SECTION 3: COMMENTS ON STRENGTHS AND LIMITATIONS** | |
| Strengths:   - Important outcome - Appropriate sample size - Robust multivariate analysis which features potentially causative confounders e.g. maternal pyrexia. (However, regression table mentions interaction between tachysystole and “complications”, but details of interaction not specified.)   Limitations:   - Concurrent assessment of uterine activity means detection of tachysystole was not blinded to other risk factors. - Significant limitation is that there was more electronic monitoring in cases which could have led to relative underdiagnosis of tachysystole in controls. (Patients without electronic monitoring would have still had monitoring of contraction frequency though and analysis done per partogram contractions.) - Contraction rate monitored per 15 rather than 10 minutes as per ACOG definition. | |

## Results of Individual Studies

Table S15 Main results of included studies. OR = odds ratio for outcome if exposure present unless otherwise stated. Unadjusted values were calculated for this study based on reported data. Adjusted values are reproduced as presented in original articles. TS = tachysystole.

| Study | Exposure Definition | Outcome Definition | Result | Authors' Conclusion |
| --- | --- | --- | --- | --- |
| Heuser et al. 2013 | >5 contractions in 10 minutes averaged over 30-minutes | 5-minute Apgar <7 | TS: 61/5363 (1.14%)  No TS: 336/44972 (0.75%)  Χ^2^= 0.002  OR: 1.53 (95% CI: 1.16-2.01) | TS “impacts foetal heart rate and neonatal morbidity” |
| Stewart et al. 2012 | Maximum number of contractions per ten minutes | 5-minute Apgar ≤3 | ≤4: 0/152 (0%)  5: 0/134 (0%)  6: 1/179 (1%)  ≥7: 0/119 (0%)  p for trend =0.86  OR if >5: N/A | TS “as currently defined when occurring remote from delivery is not associated with adverse infant outcomes.” |
|  |  | UA pH <7.1 | ≤4: 1/152 (1%)  5: 4/179 (2%)  6: 2/134 (1%)  ≥7: 6/119 (5%)  p for trend =0.06  OR if >5: 2.13 [95% CI: 0.69-6.59] |  |
|  |  | Intubation at delivery | ≤4: 1/152 (1%)  4: 0/134 (0%)  6: 0/179 (0%)  ≥7: 0/119 (0%)  P for trend =0.11  OR if >5: N/A |  |
|  | Number of single contractions lasting over 2 minutes | 5-minute Apgar ≤3 | 0: 1/495 (0.2%)  1: 0/70 (0%)  2: 0/19 (0%)  P for trend =0.69  OR if >5: N/A |  |
|  |  | UA pH <7.1 | 0:12/495 (2%)  1: 1/70 (1%)  2: 0/19 (0%)  p for trend =0.69  OR if >5: N/A |  |
|  |  | Intubation at delivery | 0: 1/495 (0.2%)  1: 0/70 (0%)  2: 0/19 (0%)  P for trend =0.69  OR if >5: N/A |  |
| Jonsson et al. 2008 | ≥6 contractions in 10 minutes | Cases: UA pH <7.05  Controls: pH≥7.05 and 5-minute Apgar ≥ 5 | Cases: 84/305 (38.9%) Controls: 43/610 (11.3%)  aOR: 5.36 (95% CI: 3.32-8.65)  Case:  TS: 84/127 (66.1%)  No TS: 132/469 (28.1%)  OR: 4.99 (95% CI: 3.28-7.58) | "A hyperactive uterine contraction pattern and oxytocin use are the most important risk factors for acidaemia at birth" |
| Mutlu Meydanli et al. 2002 | ≥6 contractions in 10 minutes for two consecutive 10-minute periods | UA pH ≤7.15 | Group numbers unavailable  RR^[[67]](#endnote-68)^: 7.1 (95% CI: 1.3–38.7)  aOR=7.3 (95% CI:1.3-40.5) | “In the absence of TS and foetal tachycardia, an uneventful delivery might be expected for women receiving 50 micrograms of intravaginal misoprostol” |
| Ahmed et al. 2016 | >5 contractions in 10 minutes averaged over a 30-minute window | Apgar score <7 at 5 minutes | TS: 2/890 (0.2%)  No TS: 14/7118 (0.2%)  Fisher’s exact p=0.897  OR: 1.14 (95% CI: 0.26-5.04) | “Uterine TS occurs in more than 10% of spontaneous labours and is… not associated with low Apgar scores.” |
| Mussi et al. 2015 | Either >5 contractions in 10 minutes over a 30-minute period or any contraction lasting >2 minutes | UA pH<7.1 or BE<-10 | No TS: 11/339(3.2%)  TS: 5/91 (5.5%)  Fisher’s exact p=0.35  OR: 1.73 (95% CI: 0.59-5.12) | “In women receiving oxytocin during labour, the… occurrence of TS” does not correlate with foetal acidaemia. |
|  |  | UA pH | UA pH r value: -0.006  p=0.8 |  |
|  |  | UA base excess | UA BE r value: 0.15  p=0.07 |  |
| Smith et al. 2014 | Any TS (>15 contractions in 30 minutes) | UA base deficit ≥10mmol/L or 5-minute Apgar ≤6 | Any TS: 11/1139 (1.0%)  No TS: 66/5095 (1.3%)  OR: 0.74 (95% CI: 0.39-1.41)  Chi square p=0.36 | "Foetuses with neonatal depression or metabolic acidaemia had no more TS than those without." |
|  | TS for 30-60 minutes |  | No TS: 66/5095 (1.3%) 30-60 minutes UT: 6/879 (0.7%)  RR: 0.5 (95% CI: 0.2-1.2) Chi square p=0.124 |  |
|  | TS for over 60 minutes |  | No UT: 66/5095 (1.3%) >60minutes UT: 5/260 (1.9%)  RR: 1.5 (95% CI: 0.6-3.7) Fisher’s exact p=0.56 |  |
| Bakker at al. 2006 | Contraction frequency (first stage) | UA pH ≤7.11 or ≥7.12 | Acidaemia: 5.0 ±0.7 Normal: 4.8 ±0.7  p=0.006 | "An UA pH 7.11 or less at birth is associated with significant more uterine activity during the first and second stage of labour." |
|  | Contraction duration (first stage) |  | Acidaemia: 87 ±9 Normal: 87 ±10  p=0.68 |  |
|  | Relaxation time (first stage) |  | Acidaemia: 51 ±23 Normal: 63 ±35  p=0.001 |  |
|  | Montevideo units (first stage) |  | Acidaemia: 261 ±86 Normal: 236 ±97  p=0.02 |  |
|  | Active planimeter units (first stage) |  | Acidaemia: 9014 ±2461 Normal: 8379 ±2740  p=0.04 |  |
|  | Contraction amplitude (first stage) |  | Acidaemia: 54 ±16 Normal: 51 ±19  p=0.165 |  |
|  | Contraction surface (first stage) |  | Acidaemia: 1875 ±555 Normal: 1798 ±600  p=0.26 |  |
| Hamilton et al. 2012 | Mean contraction rate | Metabolic acidaemia: base deficit >12  Normal: base deficit <8 | Metabolic acidaemia: 3.02 (n = 316 labours, 6,920 segments, SD: 1.77)  Normal: 2.93 (n = 33,20 labours, 66,812 segments, SD: 1.74)  p<0.0001 as quoted in paper, seems to have been calculated based on number of segments rather than labours and data does not seem to be normally distributed | “Although high contraction rates were more frequent in the metabolic acidaemia group, it was not uncommon in the normal group.” |
|  | TS (any contraction rate >5 in 10 minutes) |  | TS: 138/1353 (10.2%)  No TS: 178/2283 (7.8%)  Chi square p=0.013  OR: 1.34 (95% CI: 1.06-1.70) |  |
|  | Mean number of minutes of TS |  | Metabolic acidaemia: 28.4 (SD: 22.9)  Normal: 29.1 (SD: 28.9)  p=0.78 |  |
| Bofill et al. 2017 | Two consecutive 10-minute windows with ≥6 contractions each | 5-minute Apgar score ≤3 | ≥3 episodes: 0/131 (0%)  No episodes: 3/631 (0.5%)  P=N/A  OR: N/A | “TS was not associated with adverse perinatal outcomes 6,920 when compared to women with no TS during cervical ripening and induction of labor.” |
|  |  | UA pH <7.0 | ≥3 episodes: 3/131 (2.3%)  No episodes: 8/631 (1.3%)  Fisher’s Exact p = 0.41  OR: 1.83 (95% CI: 0.48-6.97) |  |
|  |  | Apgar score | ≥3 episodes: 9 (IQR: 8-9)  No episodes: 9 (IQR: 8-9)  P=0.502 |  |
|  |  | UA pH | ≥3 episodes: 7.22 (SD: 0.08)  No episodes: 7.22 (SD: 0.08)  P=0.435 |  |
|  |  | UA BE | ≥3 episodes: -6.81 (SD: 4.18)  No episodes: -6.57 (SD: 4.0)  P=0.535 |  |
| Palanisamy et al. 2019 | “Always” TS: >15 contractions from both 60-30 and 30-0 minutes before delivery | UA pH ≤7.1 | “Always” TS: 9/513 (1.8%)  Not “always” TS: 140/8067 (1.7%)  Chi square p =0.98  OR: 1.01 (95% CI: 0.51-2.00)  aOR (95% CI) = 1.09 (0.55-2.17) | “…cord arterial blood lactate was significantly elevated after UT during the last hour of labour… [but] was not accompanied by changes in either umbilical artery pH [or] base deficit…” |
|  |  | UA Lactate ≥4 | “Always” TS: 173/513 (33.7%)  Not “always” TS: 2032/8067 (25.2%)  Chi square p = 0.000018  aOR (95% CI) = 1.54 (1.27-1.86)  OR: 1.51 (95% CI: 1.25-1.83) |  |
|  |  | UA Base excess ≤-8 | “Always” TS: 33/513 (6.4%)  Not “always” TS: 315/8067 (3.9%)  Chi square p = 0.005  OR: 1.69 (95% CI: 1.17-2.45)  aOR (95% CI) = 1.74 (1.19-2.56) |  |
| Hayes et al. 2013 | Maximum number of pains > 7 in 15 minutes | Any neonatal encephalopathy (NE) v. No NE | TS: 87/187 (46.5%)  No TS: 120/457 (26.3%)  OR: 2.44 (95% CI: 1.71-3.48)  aOR: 2.07 (95% CI: 1.13-3.81) | "Frequency of contraction was a highly significant risk factor [for neonatal encephalopathy]" |
|  |  | Mild NE v. No NE | TS: 66/166 (39.8%)  No TS: 75/412 (18.2%)  OR: 2.97 (95% CI: 1.99-4.42)  aOR: 2.26 (95% CI: 1.14-4.49) |  |
|  |  | Moderate - severe NE v. No NE | TS: 21/121 (17.4%)  No TS: 45/382 (11.8%)  OR: 1.57 (95% CI: 0.89-2.76)  aOR: 1.23 (95% CI: 0.41-3.68) |  |

## Data Extraction Template

Modified from: *Effective Practice and Organisation of Care (EPOC). Data collection form. EPOC Resources for review authors. Oslo: Norwegian Knowledge Centre for the Health Services; 2013. Available at:* [*http://epoc.cochrane.org/epoc-specific-resources-review-authors*](http://epoc.cochrane.org/epoc-specific-resources-review-authors)

Notes on using a data extraction form: Be consistent in the order and style you use to describe the information for each included study. Record any missing information as unclear or not described, to make it clear that the information was not found in the study report(s), not that you forgot to extract it. Include any instructions and decision rules on the data collection form, or in an accompanying document. It is important to practice using the form and give training to any other authors using the form.

### General Information

| **Report title**  *(title of paper/ abstract/ report that data are extracted from)* |  |
| --- | --- |
| **Study funding source**  *(including role of funders)* |  |
| **Possible conflicts of interest**  *(for study authors)* |  |

### Eligibility

**Participants/population**

Inclusion Criteria: Term newborns delivered after spontaneous or induced labour. Exclusion criteria: Animal studies, Neonates with congenital anomalies (If any otherwise eligible studies include neonates with congenital anomalies, efforts will be made to separate this group and perform a secondary analysis.)

**Intervention(s), exposure(s)**

Give full and clear descriptions of the nature of the interventions or the exposures to be reviewed

The exposure of interest is excessive uterine activity during labour. This is not a clearly defined exposure and we

expect to find significant study heterogeneity in terms of the measurement technique and definition of the exposure. The American Congress of Obstetricians and Gynecologists defines uterine tachysystole as greater than 5 contractions in 10 minutes averaged over a thirty minute window. Studies which examine contraction rate, contraction duration, rest interval duration, resting tone, contraction strength or Montevideo units will be eligible for inclusion. There are multiple methods of measuring uterine activity - external and internal topography as well as electrical uterine myography. Studies utilising any of these methods will be eligible for inclusion.

**Comparator(s)/control**

Studies will not be excluded on the basis of their control participants, but their quality will be assessed against at least the following criteria: Controls should be term babies without congenital anomalies who experienced labour. The measurement of uterine activity and outcomes should be the same for cases as for controls.

**Types of study to be included**

Given that the dependent variable of interest is typically an exposure rather than an intervention, we anticipate that the majority of the included studies will be observational. If any interventional studies, for instance studies of oxytocin, reported the influence of uterine activity on neonatal outcomes, they will be eligible for inclusion. Studies which report both uterine activity and newborn condition as outcomes but do not comment on their relationship to each other will not be eligible for inclusion. There are no restrictions on the type of study that can be included. Specifically, retrospective and prospective studies may be included as well as case-control and cohort studies and case series. Review articles will be mined for relevant references, but will be otherwise excluded.

**Context**

Studies which include measures of intrapartum uterine activity regardless of setting will be eligible for inclusion.

**Primary outcome**

The primary outcome for this systematic review is clinical grade of neonatal encephalopathy (Sarnat grade 1-3).

Give information on timing and effect measures, as appropriate. Clinically grade of neonatal encephalopathy should ideally be determined by a consultant neurologist or neonatologist at less than 24 hours of life. However, other measures of clinical encephalopathy will also be eligible.

**Secondary outcomes**

Aside from the primary outcome, studies which measured developmental outcomes will be eligible for inclusion. Proxy measures of neonatal encephalopathy or neurodevelopmental outcome will also be acceptable.

Give information on timing and effect measures, as appropriate. The relevant proxy measures are low cord pH, elevated lactate, low APGAR scores, MRI (neonatal period or before 24 months), electroencephalography, cerebral function monitoring, and neonatal encephalopathy biomarkers.

| **Study Characteristics** | | **Review Inclusion Criteria** |
| --- | --- | --- |
| **Type of Study** | |  |
| **Participants** | |  |
| **Types of exposure** | |  |
| **Types of outcome measures** | |  |
| **Decision:** |  | |
| **Reason for exclusion** | |  |

**DO NOT PROCEED IF STUDY EXCLUDED FROM REVIEW**

### Population and setting

|  | **Description**  *Include comparative information for each group (i.e. intervention and controls) if available* |
| --- | --- |
| **Population description**  *(from which study participants are drawn e.g. spontaneous labour, induced labour)* |  |
| **Setting**  *(including location and social context)* |  |
| **Inclusion criteria** |  |
| **Exclusion criteria** |  |
| **Method/s of recruitment of participants** |  |
| **Notes:** |  |

### Methods

|  | **Descriptions as stated in report/paper** |
| --- | --- |
| **Aim of study** |  |
| **Design**  *(e.g. RCT, cohort, case-control)* |  |
| **Prospective or Retrospective?** |  |
| **Start date** |  |
| **End date** |  |
| **Duration of participation**  *(from recruitment to last follow-up)* |  |
| **Notes:** |  |

### Participants

|  | **Description as stated in report/paper** |
| --- | --- |
| **Total number** |  |
| **Clusters**  *(if applicable, no., type, no. people per cluster)* |  |
| **Baseline imbalances** |  |
| **Withdrawals and exclusions**  *(if not provided below by outcome)* |  |
| **Race/Ethnicity** |  |
| **Co-morbidities** |  |
| **Other treatment received**  *(additional to study intervention)* |  |
| **Other relevant sociodemographics** |  |
| **Subgroups measured** |  |
| **Subgroups reported** |  |
| **Notes:** |  |

### Exposure groups

|  | **Description as stated in report/paper** |
| --- | --- |
| **Group name** |  |
| **No. in group**  *(specify whether no. people or clusters)* |  |
| **Method of measurement**  *(e.g. Toco, IUPC, EM)* |  |
| **Interpretation**  *(e.g. automated or expert)* |  |
| **Duration of measurement** |  |
| **Timing**  *(e.g. time before delivery)* |  |
| **Component(s) of Uterine Activity Measured**  *(e.g. rate, relaxation time* |  |
| **Co-exposure** |  |
| **Definition of Exposure** |  |
| **Notes:** |  |

### Outcomes

|  | **Description as stated in report/paper** | |
| --- | --- | --- |
| **Outcome name** |  | |
| **Time points measured**  *(specify whether from start or end of intervention)* |  | |
| **Time points reported** |  | |
| **Outcome definition**  *(with diagnostic criteria if relevant and note whether the outcome is desirable or undesirable if this is not obvious)* |  | |
| **Person measuring/ reporting** |  | |
| **Unit of measurement**  *(if relevant)* |  | |
| **Scales: upper and lower limits**  *(indicate whether high or low score is good)* |  | |
| **Is outcome/tool validated?** | *Yes/No/Unclear* |  |
| **Notes:** |  | |

### Results

#### Dichotomous outcome

|  | | **Description as stated in report/paper** | | | |
| --- | --- | --- | --- | --- | --- |
| **Outcome** | |  | | | |
| **Subgroup** | |  | | | |
| **Results**  *Note whether:*  *Adjusted OR*  *Unadjusted* | | **Exposure Group** | | **Comparison Group** | |
|  |  | No. events | No. participants | No. events | No. participants |
|  |  |  |  |  |  |
| **No. missing participants and reasons** | |  | |  | |
| **Any other results reported** | |  | | | |
| **Unit of analysis**  *(e.g. by individuals, health professional, practice, hospital, community)* | |  | | | |
| **Statistical methods used and appropriateness of these methods**  *(e.g. adjustment for correlation)* | |  | | | |
| **Notes:** |  | | | | |

#### Continuous outcome

|  | | **Description as stated in report/paper** | | | | | |
| --- | --- | --- | --- | --- | --- | --- | --- |
| **Outcome** | |  | | | | | |
| **Subgroup** | |  | | | | | |
| **Results**  *And whether*  *Adjusted OR*  *Unadjusted* | | **Intervention** | | | **Comparison** | | |
|  |  | Mean | SD (or other variance) | No. participants | Mean | SD (or other variance) | No. participants |
|  |  |  |  |  |  |  |  |
| **No. missing participants and reasons** | |  | |  | | | |
| **Any other results reported** | |  | | | | | |
| **Unit of analysis**  *(e.g. by individuals, health professional, practice, hospital, community)* | |  | | | | | |
| **Statistical methods used and appropriateness of these methods**  *(e.g. adjustment for correlation)* | |  | | | | | |
| **Notes:** |  | | | | | | |

### Applicability

| **Have important populations been excluded from the study?**  *(consider disadvantaged populations, and possible differences in the intervention effect)* | | *Yes/No/Unclear* |  |
| --- | --- | --- | --- |
| **Does the study directly address the review question?**  *(any issues of partial or indirect applicability)* | | *Yes/No/Unclear* |  |
| **Notes:** |  | | |

### Other information

|  | | **Description as stated in report/paper** |
| --- | --- | --- |
| **Key conclusions of study authors** | |  |
| **References to other relevant studies** | |  |
| **Notes:** |  | |

# Supplemental Material Bibliography

1. Kunz MK, Loftus RJ, Nichols AA. Incidence of Uterine Tachysystole in Women Induced with Oxytocin. Journal of Obstetric, Gynecologic & Neonatal Nursing. 2013;42(1):12-8.

1. TS averaged over 60 minutes, as opposed to ACOG definition of over 30. [↑](#endnote-ref-2)
2. Patients selected in unbiased manner from population, but different based on associations with tachysystole. [↑](#endnote-ref-3)
3. All eligible patients included. [↑](#endnote-ref-4)
4. Impossible to ensure but unlikely to affect measurement of outcome. [↑](#endnote-ref-5)
5. Unlikely that knowledge of uterine activity had significant effect on assessment of outcome. However, assessment of uterine activity was not blinded to knowledge of other factors e.g. foetal heart rate and that may have had an effect. [↑](#endnote-ref-6)
6. Data on the reliability of the identification of tachysystole is lacking. One study employing retrospective analysis of foetal strips reported a Pearson correlation for the presence/absence of tachysystole of 86.3%. ([1](#_ENREF_1)) [↑](#endnote-ref-7)
7. Yes, but not for outcomes included in this study. [↑](#endnote-ref-8)
8. Baseline characteristics reported, but not divided by level of uterine activity. [↑](#endnote-ref-9)
9. All eligible patients included. [↑](#endnote-ref-10)
10. Only patients with no decelerations on foetal heart tracing prior to induction were included. [↑](#endnote-ref-11)
11. No patients lot to follow up, but no data presented on exclusions due to lack of data. [↑](#endnote-ref-12)
12. Impossible to ensure but unlikely to affect measurement of outcome. Assessment of exposure was made blind to outcome status. [↑](#endnote-ref-13)
13. Unlikely that knowledge of uterine activity had significant effect on assessment of outcome. [↑](#endnote-ref-14)
14. Data on the reliability of the identification of tachysystole is lacking. One study employing retrospective analysis of foetal strips reported a Pearson correlation for the presence/absence of tachysystole of 86.3%. ([1](#_ENREF_1)) [↑](#endnote-ref-15)
15. Patients selected in unbiased manner from population, but different based on associations with tachysystole. [↑](#endnote-ref-16)
16. Yes, but division not based on tachysystole. [↑](#endnote-ref-17)
17. Yes, main component of outcome measure is objective i.e. UA pH. [↑](#endnote-ref-18)
18. Measurement of UA pH can be complicated by inadvertent sampling of UV [↑](#endnote-ref-19)
19. Controls matched to cases for parity but the appropriateness of this is questionable. Usual confounders not controlled for in multivariate analysis. [↑](#endnote-ref-20)
20. Yes, but provided unadjusted intervals not used as based on risk, not odds. [↑](#endnote-ref-21)
21. Group with adverse outcome and negative outcome were broadly similar. Characteristics not reported divided by tachysystole status. [↑](#endnote-ref-22)
22. All eligible patients included. [↑](#endnote-ref-23)
23. Data on the reliability of the identification of tachysystole is lacking. One study employing retrospective analysis of foetal strips reported a Pearson correlation for the presence/absence of tachysystole of 86.3%. ([1](#_ENREF_1)) [↑](#endnote-ref-24)
24. Details of logistic regression analysis not reported [↑](#endnote-ref-25)
25. Yes, but group numbers not reported for eligible outcome. [↑](#endnote-ref-26)
26. Yes, but need to be mindful of particular characteristics of this group i.e. only induction with “unfavourable” cervix. [↑](#endnote-ref-27)
27. Patients selected in unbiased manner from population, but different based on associations with tachysystole. [↑](#endnote-ref-28)
28. All eligible patients included. [↑](#endnote-ref-29)
29. Data on the reliability of the identification of tachysystole is lacking. This study used concurrent assessment by attending healthcare staff, but a decision support tool was in use and may have influenced interpretation. No reliability or accuracy data on the decision supports tool’s identification of tachysystole was reported. ([1](#_ENREF_1)) [↑](#endnote-ref-30)
30. Multivariate logistic regression with tachysystole as the outcome was performed with possible determinants of uterine activity as the independent variables. No adjusted values were presented for neonatal outcomes. [↑](#endnote-ref-31)
31. Yes, but need to be mindful of particular characteristics of this group i.e. only spontaneous unaugmented labours included. [↑](#endnote-ref-32)
32. Patients selected in unbiased manner from population, but different based on associations with tachysystole. Group characteristics as divided by tachysystole status were not described. [↑](#endnote-ref-33)
33. All eligible patients included. [↑](#endnote-ref-34)
34. Data on the reliability of the identification of tachysystole is lacking. This study used concurrent assessment by attending healthcare staff, but a decision support tool was in use and may have influenced interpretation. No reliability or accuracy data on the decision supports tool’s identification of tachysystole was reported. ([1](#_ENREF_1)) There is no data to support the equivalency of uterine tachysystole and hypertonus. [↑](#endnote-ref-35)
35. Multivariate analysis not detailed. Not clear that uterine activity was included in the model. [↑](#endnote-ref-36)
36. Yes, but need to be mindful of particular characteristics of this group i.e. only cases receiving oxytocin during labour were included. [↑](#endnote-ref-37)
37. Patients selected in unbiased manner from population, but different based on associations with tachysystole. [↑](#endnote-ref-38)
38. All eligible patients included. [↑](#endnote-ref-39)
39. Possible but unlikely that knowledge of uterine activity affected assessment of Apgar score [↑](#endnote-ref-40)
40. Data on the reliability of the identification of tachysystole is lacking. This study used concurrent assessment by attending healthcare staff, but a decision support tool was in use and may have influenced interpretation. No reliability or accuracy data on the decision supports tool’s identification of tachysystole was reported. ([1](#_ENREF_1)) [↑](#endnote-ref-41)
41. No multivariate analysis, but univariate result was not statistically significant. [↑](#endnote-ref-42)
42. Patients selected in unbiased manner from population, but different based on associations with tachysystole. Group characteristics were described divided by pH status rather than tachysystole. [↑](#endnote-ref-43)
43. All eligible patients included. [↑](#endnote-ref-44)
44. Data on the reliability of the algorithm used to interpret the IUPC recordings was not reported. [↑](#endnote-ref-45)
45. No multivariate analysis performed [↑](#endnote-ref-46)
46. Yes, but need to be mindful that this group all had an indication for IUPC insertion, although IUPCs were more liberally employed in this population. [↑](#endnote-ref-47)
47. Patients selected in unbiased manner from population, but different based on associations with tachysystole. Group characteristics were described divided by pH status rather than tachysystole. Furthermore, babies with signs of encephalopathy were excluded but no data on number of exclusions or on the breakdown of exclusions per tachysystole group. [↑](#endnote-ref-48)
48. All eligible patients included. [↑](#endnote-ref-49)
49. Data on the reliability of the algorithm for the detection and classification of foetal heart rate decelerations was published, but reliability for detection of uterine contractions not mentioned in paper. [↑](#endnote-ref-50)
50. No multivariate analysis performed [↑](#endnote-ref-51)
51. Yes, but need to be mindful that this group all had an indication for IUPC insertion, although IUPCs were more liberally employed in this population. [↑](#endnote-ref-52)
52. Patients selected in unbiased manner from population, but different based on associations with tachysystole. Tachysystole group were younger and had less maternal obesity and hypertension. [↑](#endnote-ref-53)
53. All eligible patients included. [↑](#endnote-ref-54)
54. Data on the reliability of the algorithm for the detection and classification of foetal heart rate decelerations was published, but reliability for detection of uterine contractions not mentioned in paper. [↑](#endnote-ref-55)
55. Multivariate analysis performed, but significant confounders not reported or included in model. [↑](#endnote-ref-56)
56. Patients selected in unbiased manner from population, but different based on associations with tachysystole. Tachysystole group were younger and had higher rates of nulliparity, post-maturity, pre-eclampsia, prolonged rupture of membranes, meconium-stained liquor and non-reassuring foetal heart rate traces. [↑](#endnote-ref-57)
57. Some data on recruitment reported in reports of individual trials, but not according to TS status. [↑](#endnote-ref-58)
58. Knowledge of TS status may have impacted assessment of Apgar score, but unlikely to have had a significant effect if any. [↑](#endnote-ref-59)
59. Analysis was performed by a single interpreter, but no data on reliability of interpretation presented. Blinding of assessment was not reported. [↑](#endnote-ref-60)
60. No multivariate analysis [↑](#endnote-ref-61)
61. Patients selected in unbiased manner from population, but different based on associations with tachysystole. [↑](#endnote-ref-62)
62. Yes, but division based on encephalopathy status, not tachysystole. [↑](#endnote-ref-63)
63. Unlikely that knowledge of TS per se would have influenced diagnosis of neonatal encephalopathy, but other risk factors i.e. foetal heart rate patterns could have influenced ascertainment of both. [↑](#endnote-ref-64)
64. Data on the reliability of the identification of tachysystole is lacking. This study used concurrent assessment by attending healthcare staff, but a decision support tool was in use and may have influenced interpretation. No reliability or accuracy data on the decision supports tool’s identification of tachysystole was reported. ([1](#_ENREF_1)) [↑](#endnote-ref-65)
65. Yes, but tachysystole was dependant rather than independent variable for analysis [↑](#endnote-ref-66)
66. Based mainly on monitoring differential between controls and cases and the lack of blinded assessment of uterine activity. More of the cases had electronic foetal monitoring. No data on how this impacted ascertainment of tachysystole. [↑](#endnote-ref-67)
67. Underlying data not presented, only RR and CI. RR as reported by authors. [↑](#endnote-ref-68)
